# Supplementary figures and images for: CHARM: COVID-19 Health Action Response for Marines–Association of antigen-specific interferon-gamma and IL2 responses with asymptomatic and symptomatic infections after a positive qPCR SARS-CoV-2 test
Source: PLoS One. 2022 Apr 7;17(4):e0266691. doi: 10.1371/journal.pone.0266691 (PMC8989306; doi:10.1371/journal.pone.0266691)

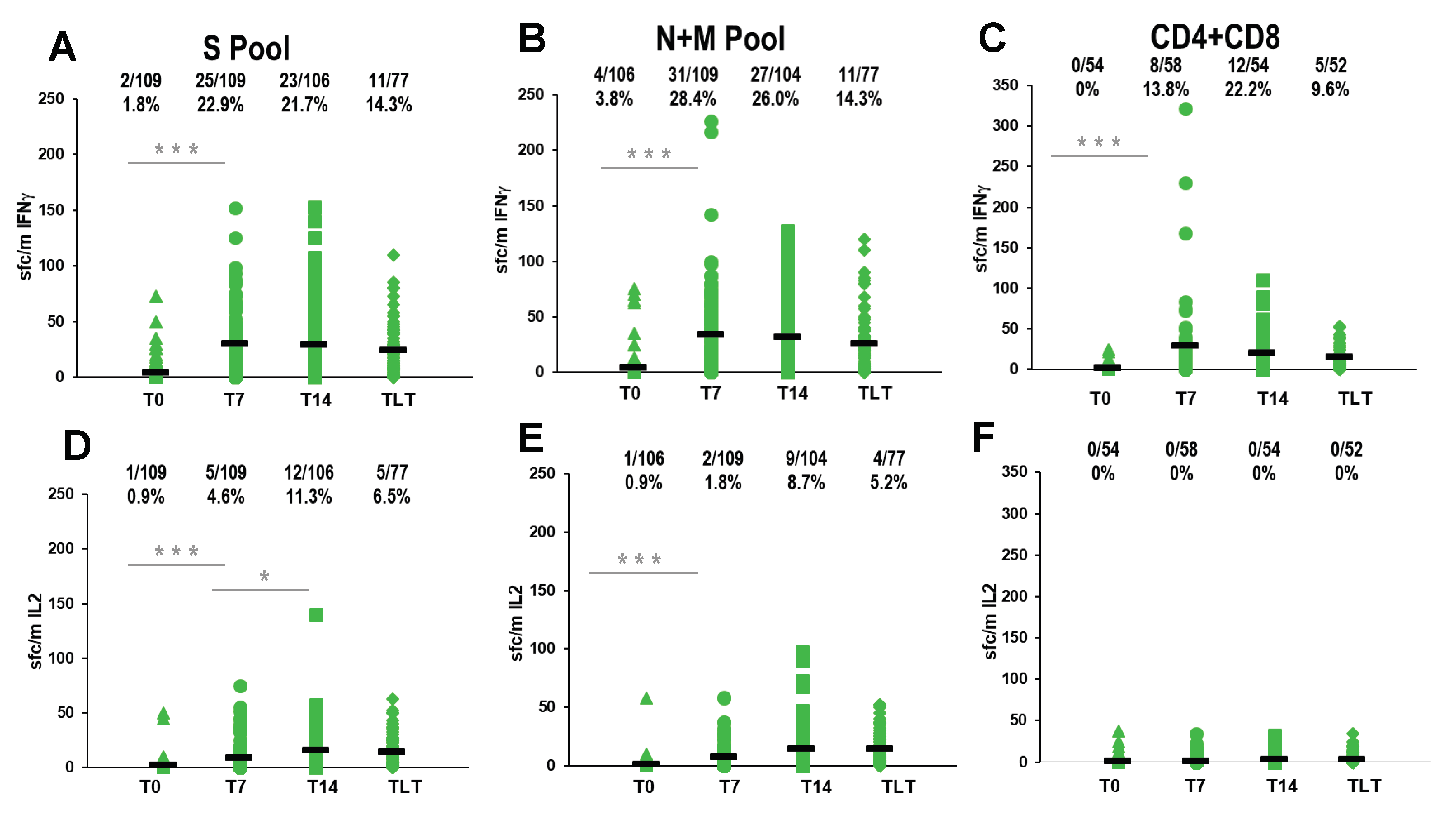

Supplement: S1 Fig — *** p = <0.001; * p = <0.05. The numbers of samples at each time point were based on the numbers of available samples among the 124 asymptomatic participants. Immune responses (sfc/m) and percent positive participants are shown before the first positive qPCR test (T0), 7 days (T7), 14 days (T14) and long-term (TLT) after that first positive PCR test (infection). Immune responses to S, N+M and CD4+CD8 pools were significantly (p = <0.001) higher at T7, and were not significantly different between T7, T14 and TLT after infection; the exception was the significant rise in IL2 responses to S pool between 7d and 14d after infection. There were no positive IL2 responses to the CD4+CD8 pool. (TIFF) [file pone.0266691.s001.tiff]

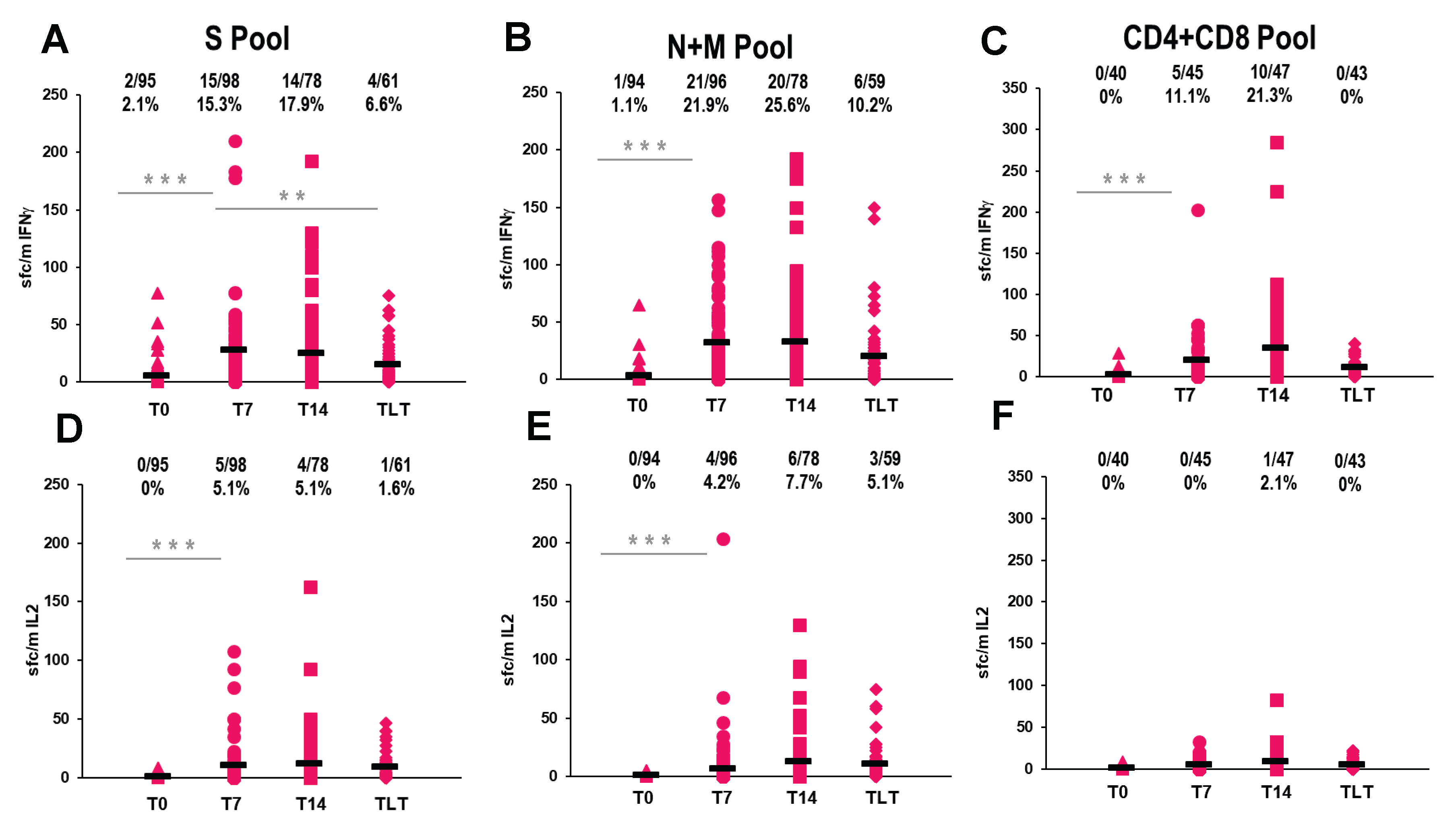

Supplement: S2 Fig — ***p = <0.001; **p = <0.01. The numbers of samples at each time point were based on the numbers of available samples among the 105 symptomatic participants. Immune responses (sfc/m) and percent positive participants are shown before the first positive PCR test (T0), and 7 days (T7), 14 days (T14) and long-term (TLT) after that first positive PCR test (infection). Immune responses to S, N+M and CD4+CD8 pools were significantly (p = <0.001) higher at T7, and IFN-γ responses to S protein significantly dropped by TLT. IL2 responses to the CD4+CD8 pool were absent, except in one participant at 14d after infection. (TIFF) [file pone.0266691.s002.tiff]
