# Supplementary material for: CHARM: COVID-19 Health Action Response for Marines–Association of antigen-specific interferon-gamma and IL2 responses with asymptomatic and symptomatic infections after a positive qPCR SARS-CoV-2 test
Source: PLoS One. 2022 Apr 7;17(4):e0266691. doi: 10.1371/journal.pone.0266691 (PMC8989306; doi:10.1371/journal.pone.0266691)
Supplement: S1 Table — This 181-peptide array spans the spike (S) glycoprotein of the USA-WA1/2020 strain of SARS-CoV-2 (GenPept. QH060594). (DOCX) [file pone.0266691.s003.docx]

**Table S1. S protein peptides**

| **Peptide** | **Length** | **Sequence** |
| --- | --- | --- |
| 1 of 181 | 17 | 1-MFVFLVLLPLVSSQCVN-17 |
| 2 of 181 | 17 | 8-LPLVSSQCVNLTTRTQL-24 |
| 3 of 181 | 17 | 15-CVNLTTRTQLPPAYTNS-31 |
| 4 of 181 | 17 | 22-TQLPPAYTNSFTRGVYY-38 |
| 5 of 181 | 17 | 29-TNSFTRGVYYPDKVFRS-45 |
| 6 of 181 | 17 | 36-VYYPDKVFRSSVLHSTQ-52 |
| 7 of 181 | 17 | 43-FRSSVLHSTQDLFLPFF-59 |
| 8 of 181 | 17 | 50-STQDLFLPFFSNVTWFH-66 |
| 9 of 181 | 17 | 57-PFFSNVTWFHAIHVSGT-73 |
| 10 of 181 | 17 | 64-WFHAIHVSGTNGTKRFD-80 |
| 11 of 181 | 17 | 71-SGTNGTKRFDNPVLPFN-87 |
| 12 of 181 | 17 | 78-RFDNPVLPFNDGVYFAS-94 |
| 13 of 181 | 17 | 85-PFNDGVYFASTEKSNII-101 |
| 14 of 181 | 17 | 92-FASTEKSNIIRGWIFGT-108 |
| 15 of 181 | 17 | 99-NIIRGWIFGTTLDSKTQ-115 |
| 16 of 181 | 17 | 106-FGTTLDSKTQSLLIVNN-122 |
| 17 of 181 | 17 | 113-KTQSLLIVNNATNVVIK-129 |
| 18 of 181 | 17 | 120-VNNATNVVIKVCEFQFC-136 |
| 19 of 181 | 17 | 127-VIKVCEFQFCNDPFLGV-143 |
| 20 of 181 | 17 | 134-QFCNDPFLGVYYHKNNK-150 |
| 21 of 181 | 17 | 141-LGVYYHKNNKSWMESEF-157 |
| 22 of 181 | 17 | 148-NNKSWMESEFRVYSSAN-164 |
| 23 of 181 | 17 | 155-SEFRVYSSANNCTFEYV-171 |
| 24 of 181 | 17 | 162-SANNCTFEYVSQPFLMD-178 |
| 25 of 181 | 17 | 169-EYVSQPFLMDLEGKQGN-185 |
| 26 of 181 | 17 | 176-LMDLEGKQGNFKNLREF-192 |
| 27 of 181 | 17 | 183-QGNFKNLREFVFKNIDG-199 |
| 28 of 181 | 17 | 190-REFVFKNIDGYFKIYSK-206 |
| 29 of 181 | 17 | 197-IDGYFKIYSKHTPINLV-213 |
| 30 of 181 | 17 | 204-YSKHTPINLVRDLPQGF-220 |
| 31 of 181 | 17 | 211-NLVRDLPQGFSALEPLV-227 |
| 32 of 181 | 17 | 218-QGFSALEPLVDLPIGIN-234 |
| 33 of 181 | 17 | 225-PLVDLPIGINITRFQTL-241 |
| 34 of 181 | 17 | 232-GINITRFQTLLALHRSY-248 |
| 35 of 181 | 17 | 239-QTLLALHRSYLTPGDSS-255 |
| 36 of 181 | 17 | 246-RSYLTPGDSSSGWTAGA-262 |
| 37 of 181 | 17 | 253-DSSSGWTAGAAAYYVGY-269 |
| 38 of 181 | 17 | 260-AGAAAYYVGYLQPRTFL-276 |
| 39 of 181 | 17 | 267-VGYLQPRTFLLKYNENG-283 |
| 40 of 181 | 17 | 274-TFLLKYNENGTITDAVD-290 |
| 41 of 181 | 17 | 281-ENGTITDAVDCALDPLS-297 |
| 42 of 181 | 17 | 288-AVDCALDPLSETKCTLK-304 |
| 43 of 181 | 17 | 295-PLSETKCTLKSFTVEKG-311 |
| 44 of 181 | 17 | 302-TLKSFTVEKGIYQTSNF-318 |
| 45 of 181 | 17 |  |
| 46 of 181 | 17 | 316-SNFRVQPTESIVRFPNI-332 |
| 47 of 181 | 17 | 323-TESIVRFPNITNLCPFG-339 |
| 48 of 181 | 17 | 330-PNITNLCPFGEVFNATR-346 |
| 49 of 181 | 17 | 337-PFGEVFNATRFASVYAW-353 |
| 50 of 181 | 17 | 344-ATRFASVYAWNRKRISN-360 |
| 51 of 181 | 17 | 351-YAWNRKRISNCVADYSV-367 |
| 52 of 181 | 17 | 358-ISNCVADYSVLYNSASF-374 |
| 53 of 181 | 17 | 365-YSVLYNSASFSTFKCYG-381 |
| 54 of 181 | 17 | 372-ASFSTFKCYGVSPTKLN-388 |
| 55 of 181 | 17 | 379-CYGVSPTKLNDLCFTNV-395 |
| 56 of 181 | 17 | 386-KLNDLCFTNVYADSFVI-402 |
| 57 of 181 | 17 | 393-TNVYADSFVIRGDEVRQ-409 |
| 58 of 181 | 17 | 400-FVIRGDEVRQIAPGQTG-416 |
| 59 of 181 | 17 | 407-VRQIAPGQTGKIADYNY-423 |
| 60 of 181 | 17 | 414-QTGKIADYNYKLPDDFT-430 |
| 61 of 181 | 17 | 421-YNYKLPDDFTGCVIAWN-437 |
| 62 of 181 | 17 | 428-DFTGCVIAWNSNNLDSK-444 |
| 63 of 181 | 17 | 435-AWNSNNLDSKVGGNYNY-451 |
| 64 of 181 | 17 | 442-DSKVGGNYNYLYRLFRK-458 |
| 65 of 181 | 17 | 449-YNYLYRLFRKSNLKPFE-465 |
| 66 of 181 | 17 | 456-FRKSNLKPFERDISTEI-472 |
| 67 of 181 | 17 | 463-PFERDISTEIYQAGSTP-479 |
| 68 of 181 | 17 | 470-TEIYQAGSTPCNGVEGF-486 |
| 69 of 181 | 17 | 477-STPCNGVEGFNCYFPLQ-493 |
| 70 of 181 | 17 | 484-EGFNCYFPLQSYGFQPT-500 |
| 71 of 181 | 17 | 491-PLQSYGFQPTNGVGYQP-507 |
| 72 of 181 | 17 | 498-QPTNGVGYQPYRVVVLS-514 |
| 73 of 181 | 17 | 505-YQPYRVVVLSFELLHAP-521 |
| 74 of 181 | 17 | 512-VLSFELLHAPATVCGPK-528 |
| 75 of 181 | 17 | 519-HAPATVCGPKKSTNLVK-535 |
| 76 of 181 | 17 | 526-GPKKSTNLVKNKCVNFN-542 |
| 77 of 181 | 17 | 533-LVKNKCVNFNFNGLTGT-549 |
| 78 of 181 | 17 | 540-NFNFNGLTGTGVLTESN-556 |
| 79 of 181 | 17 | 547-TGTGVLTESNKKFLPFQ-563 |
| 80 of 181 | 17 | 554-ESNKKFLPFQQFGRDIA-570 |
| 81 of 181 | 17 | 561-PFQQFGRDIADTTDAVR-577 |
| 82 of 181 | 17 | 568-DIADTTDAVRDPQTLEI-584 |
| 83 of 181 | 17 | 575-AVRDPQTLEILDITPCS-591 |
| 84 of 181 | 17 | 582-LEILDITPCSFGGVSVI-598 |
| 85 of 181 | 17 | 589-PCSFGGVSVITPGTNTS-605 |
| 86 of 181 | 17 | 596-SVITPGTNTSNQVAVLY-612 |
| 87 of 181 | 17 | 603-NTSNQVAVLYQDVNCTE-619 |
| 88 of 181 | 17 | 610-VLYQDVNCTEVPVAIHA-626 |
| 89 of 181 | 17 | 617-CTEVPVAIHADQLTPTW-633 |
| 90 of 181 | 17 | 624-IHADQLTPTWRVYSTGS-640 |
| 91 of 181 | 17 | 631-PTWRVYSTGSNVFQTRA-647 |
| 92 of 181 | 17 | 638-TGSNVFQTRAGCLIGAE-654 |
| 93 of 181 | 17 | 645-TRAGCLIGAEHVNNSYE-661 |
| 94 of 181 | 17 | 652-GAEHVNNSYECDIPIGA-668 |
| 95 of 181 | 17 | 659-SYECDIPIGAGICASYQ-675 |
| 96 of 181 | 17 | 666-IGAGICASYQTQTNSPR-682 |
| 97 of 181 | 17 | 673-SYQTQTNSPRRARSVAS-689 |
| 98 of 181 | 17 | 680-SPRRARSVASQSIIAYT-696 |
| 99 of 181 | 17 | 687-VASQSIIAYTMSLGAEN-703 |
| 100 of 181 | 17 | 694-AYTMSLGAENSVAYSNN-710 |
| 101 of 181 | 17 | 701-AENSVAYSNNSIAIPTN-717 |
| 102 of 181 | 17 | 708-SNNSIAIPTNFTISVTT-724 |
| 103 of 181 | 17 | 715-PTNFTISVTTEILPVSM-731 |
| 104 of 181 | 17 | 722-VTTEILPVSMTKTSVDC-738 |
| 105 of 181 | 17 | 729-VSMTKTSVDCTMYICGD-745 |
| 106 of 181 | 17 | 736-VDCTMYICGDSTECSNL-752 |
| 107 of 181 | 17 | 743-CGDSTECSNLLLQYGSF-759 |
| 108 of 181 | 17 | 750-SNLLLQYGSFCTQLNRA-766 |
| 109 of 181 | 17 | 757-GSFCTQLNRALTGIAVE-773 |
| 110 of 181 | 17 | 764-NRALTGIAVEQDKNTQE-780 |
| 111 of 181 | 17 | 771-AVEQDKNTQEVFAQVKQ-787 |
| 112 of 181 | 17 | 778-TQEVFAQVKQIYKTPPI-794 |
| 113 of 181 | 17 | 785-VKQIYKTPPIKDFGGFN-801 |
| 114 of 181 | 17 | 792-PPIKDFGGFNFSQILPD-808 |
| 115 of 181 | 17 | 799-GFNFSQILPDPSKPSKR-815 |
| 116 of 181 | 17 | 806-LPDPSKPSKRSFIEDLL-822 |
| 117 of 181 | 17 | 813-SKRSFIEDLLFNKVTLA-829 |
| 118 of 181 | 17 | 820-DLLFNKVTLADAGFIKQ-836 |
| 119 of 181 | 17 | 827-TLADAGFIKQYGDCLGD-843 |
| 120 of 181 | 17 | 834-IKQYGDCLGDIAARDLI-850 |
| 121 of 181 | 17 | 841-LGDIAARDLICAQKFNG-857 |
| 122 of 181 | 17 | 848-DLICAQKFNGLTVLPPL-864 |
| 123 of 181 | 17 | 855-FNGLTVLPPLLTDEMIA-871 |
| 124 of 181 | 17 | 862-PPLLTDEMIAQYTSALL-878 |
| 125 of 181 | 17 | 869-MIAQYTSALLAGTITSG-885 |
| 126 of 181 | 17 | 876-ALLAGTITSGWTFGAGA-892 |
| 127 of 181 | 17 | 883-TSGWTFGAGAALQIPFA-899 |
| 128 of 181 | 17 | 890-AGAALQIPFAMQMAYRF-906 |
| 129 of 181 | 17 | 897-PFAMQMAYRFNGIGVTQ-913 |
| 130 of 181 | 17 | 904-YRFNGIGVTQNVLYENQ-920 |
| 131 of 181 | 17 | 911-VTQNVLYENQKLIANQF-927 |
| 132 of 181 | 17 | 918-ENQKLIANQFNSAIGKI-934 |
| 133 of 181 | 17 | 925-NQFNSAIGKIQDSLSST-941 |
| 134 of 181 | 17 | 932-GKIQDSLSSTASALGKL-948 |
| 135 of 181 | 17 | 939-SSTASALGKLQDVVNQN-955 |
| 136 of 181 | 17 | 946-GKLQDVVNQNAQALNTL-962 |
| 137 of 181 | 17 | 953-NQNAQALNTLVKQLSSN-969 |
| 138 of 181 | 17 | 960-NTLVKQLSSNFGAISSV-976 |
| 139 of 181 | 17 | 967-SSNFGAISSVLNDILSR-983 |
| 140 of 181 | 17 | 974-SSVLNDILSRLDKVEAE-990 |
| 141 of 181 | 17 | 981-LSRLDKVEAEVQIDRLI-997 |
| 142 of 181 | 17 | 988-EAEVQIDRLITGRLQSL-1004 |
| 143 of 181 | 17 | 995-RLITGRLQSLQTYVTQQ-1011 |
| 144 of 181 | 17 | 1002-QSLQTYVTQQLIRAAEI-1018 |
| 145 of 181 | 17 | 1009-TQQLIRAAEIRASANLA-1025 |
| 146 of 181 | 17 | 1016-AEIRASANLAATKMSEC-1032 |
| 147 of 181 | 17 | 1023-NLAATKMSECVLGQSKR-1039 |
| 148 of 181 | 17 | 1030-SECVLGQSKRVDFCGKG-1046 |
| 149 of 181 | 17 | 1037-SKRVDFCGKGYHLMSFP-1053 |
| 150 of 181 | 17 | 1044-GKGYHLMSFPQSAPHGV-1060 |
| 151 of 181 | 17 | 1051-SFPQSAPHGVVFLHVTY-1067 |
| 152 of 181 | 17 | 1058-HGVVFLHVTYVPAQEKN-1074 |
| 153 of 181 | 17 | 1065-VTYVPAQEKNFTTAPAI-1081 |
| 154 of 181 | 17 | 1072-EKNFTTAPAICHDGKAH-1088 |
| 155 of 181 | 17 | 1079-PAICHDGKAHFPREGVF-1095 |
| 156 of 181 | 17 | 1086-KAHFPREGVFVSNGTHW-1102 |
| 157 of 181 | 17 | 1093-GVFVSNGTHWFVTQRNF-1109 |
| 158 of 181 | 17 | 1100-THWFVTQRNFYEPQIIT-1116 |
| 159 of 181 | 17 | 1107-RNFYEPQIITTDNTFVS-1123 |
| 160 of 181 | 17 | 1114-IITTDNTFVSGNCDVVI-1130 |
| 161 of 181 | 17 | 1121-FVSGNCDVVIGIVNNTV-1137 |
| 162 of 181 | 17 | 1128-VVIGIVNNTVYDPLQPE-1144 |
| 163 of 181 | 17 | 1135-NTVYDPLQPELDSFKEE-1151 |
| 164 of 181 | 17 | 1142-QPELDSFKEELDKYFKN-1158 |
| 165 of 181 | 17 | 1149-KEELDKYFKNHTSPDVD-1165 |
| 166 of 181 | 17 | 1156-FKNHTSPDVDLGDISGI-1172 |
| 167 of 181 | 17 | 1163-DVDLGDISGINASVVNI-1179 |
| 168 of 181 | 17 | 1170-SGINASVVNIQKEIDRL-1186 |
| 169 of 181 | 17 | 1177-VNIQKEIDRLNEVAKNL-1193 |
| 170 of 181 | 17 | 1184-DRLNEVAKNLNESLIDL-1200 |
| 171 of 181 | 17 | 1191-KNLNESLIDLQELGKYE-1207 |
| 172 of 181 | 17 | 1198-IDLQELGKYEQYIKWPW-1214 |
| 173 of 181 | 17 | 1205-KYEQYIKWPWYIWLGFI-1221 |
| 174 of 181 | 17 | 1212-WPWYIWLGFIAGLIAIV-1228 |
| 175 of 181 | 17 | 1219-GFIAGLIAIVMVTIMLC-1235 |
| 176 of 181 | 17 | 1226-AIVMVTIMLCCMTSCCS-1242 |
| 177 of 181 | 17 | 1233-MLCCMTSCCSCLKGCCS-1249 |
| 178 of 181 | 17 | 1240-CCSCLKGCCSCGSCCKF-1256 |
| 179 of 181 | 17 | 1247-CCSCGSCCKFDEDDSEP-1263 |
| 180 of 181 | 17 | 1254-CKFDEDDSEPVLKGVKL-1270 |
| 181 of 181 | 13 | 1261-SEPVLKGVKLHYT-1273 |

This 181-peptide array spans the spike (S) glycoprotein of the USA-WA1/2020 strain of SARS-CoV-2 (GenPept. QH060594).
