# Supplementary material for: CHARM: COVID-19 Health Action Response for Marines–Association of antigen-specific interferon-gamma and IL2 responses with asymptomatic and symptomatic infections after a positive qPCR SARS-CoV-2 test
Source: PLoS One. 2022 Apr 7;17(4):e0266691. doi: 10.1371/journal.pone.0266691 (PMC8989306; doi:10.1371/journal.pone.0266691)
Supplement: S2 Table — This 59-peptide array spans the nucleocapsid (N) protein of the USA-WA1/2020 strain of SARS-CoV-2 (GenPept. QH060601). (DOCX) [file pone.0266691.s004.docx]

**Table S2. N protein peptides**

| **Peptide** | **Length** | **Sequence** |
| --- | --- | --- |
| 1 of 59 | 17 | 1-MSDNGPQNQRNAPRITF-17 |
| 2 of 59 | 17 | 8-NQRNAPRITFGGPSDST-24 |
| 3 of 59 | 17 | 15-ITFGGPSDSTGSNQNGE-31 |
| 4 of 59 | 17 | 22-DSTGSNQNGERSGARSK-38 |
| 5 of 59 | 17 | 29-NGERSGARSKQRRPQGL-45 |
| 6 of 59 | 17 | 36-RSKQRRPQGLPNNTASW-52 |
| 7 of 59 | 17 | 43-QGLPNNTASWFTALTQH-59 |
| 8 of 59 | 17 | 50-ASWFTALTQHGKEDLKF-66 |
| 9 of 59 | 17 | 57-TQHGKEDLKFPRGQGVP-73 |
| 10 of 59 | 17 | 64-LKFPRGQGVPINTNSSP-80 |
| 11 of 59 | 17 | 71-GVPINTNSSPDDQIGYY-87 |
| 12 of 59 | 17 | 78-SSPDDQIGYYRRATRRI-94 |
| 13 of 59 | 17 | 85-GYYRRATRRIRGGDGKM-101 |
| 14 of 59 | 17 | 92-RRIRGGDGKMKDLSPRW-108 |
| 15 of 59 | 17 | 99-GKMKDLSPRWYFYYLGT-115 |
| 16 of 59 | 17 | 106-PRWYFYYLGTGPEAGLP-122 |
| 17 of 59 | 17 | 113-LGTGPEAGLPYGANKDG-129 |
| 18 of 59 | 17 | 120-GLPYGANKDGIIWVATE-136 |
| 19 of 59 | 17 | 127-KDGIIWVATEGALNTPK-143 |
| 20 of 59 | 17 | 134-ATEGALNTPKDHIGTRN-150 |
| 21 of 59 | 17 | 141-TPKDHIGTRNPANNAAI-157 |
| 22 of 59 | 17 | 148-TRNPANNAAIVLQLPQG-164 |
| 23 of 59 | 17 | 155-AAIVLQLPQGTTLPKGF-171 |
| 24 of 59 | 17 | 162-PQGTTLPKGFYAEGSRG-178 |
| 25 of 59 | 17 | 169-KGFYAEGSRGGSQASSR-185 |
| 26 of 59 | 17 | 176-SRGGSQASSRSSSRSRN-192 |
| 27 of 59 | 17 | 183-SSRSSSRSRNSSRNSTP-199 |
| 28 of 59 | 17 | 190-SRNSSRNSTPGSSRGTS-206 |
| 29 of 59 | 17 | 197-STPGSSRGTSPARMAGN-213 |
| 30 of 59 | 17 | 204-GTSPARMAGNGGDAALA-220 |
| 31 of 59 | 17 | 211-AGNGGDAALALLLLDRL-227 |
| 32 of 59 | 17 | 218-ALALLLLDRLNQLESKM-234 |
| 33 of 59 | 17 | 225-DRLNQLESKMSGKGQQQ-241 |
| 34 of 59 | 17 | 232-SKMSGKGQQQQGQTVTK-248 |
| 35 of 59 | 17 | 239-QQQQGQTVTKKSAAEAS-255 |
| 36 of 59 | 17 | 246-VTKKSAAEASKKPRQKR-262 |
| 37 of 59 | 17 | 253-EASKKPRQKRTATKAYN-269 |
| 38 of 59 | 17 | 260-QKRTATKAYNVTQAFGR-276 |
| 39 of 59 | 17 | 267-AYNVTQAFGRRGPEQTQ-283 |
| 40 of 59 | 17 | 274-FGRRGPEQTQGNFGDQE-290 |
| 41 of 59 | 17 | 281-QTQGNFGDQELIRQGTD-297 |
| 42 of 59 | 17 | 288-DQELIRQGTDYKHWPQI-304 |
| 43 of 59 | 17 | 295-GTDYKHWPQIAQFAPSA-311 |
| 44 of 59 | 17 | 302-PQIAQFAPSASAFFGMS-318 |
| 45 of 59 | 17 | 309-PSASAFFGMSRIGMEVT-325 |
| 46 of 59 | 17 | 316-GMSRIGMEVTPSGTWLT-332 |
| 47 of 59 | 17 | 323-EVTPSGTWLTYTGAIKL-339 |
| 48 of 59 | 17 | 330-WLTYTGAIKLDDKDPNF-346 |
| 49 of 59 | 17 | 337-IKLDDKDPNFKDQVILL-353 |
| 50 of 59 | 17 | 344-PNFKDQVILLNKHIDAY-360 |
| 51 of 59 | 17 | 351-ILLNKHIDAYKTFPPTE-367 |
| 52 of 59 | 17 | 358-DAYKTFPPTEPKKDKKK-374 |
| 53 of 59 | 17 | 365-PTEPKKDKKKKADETQA-381 |
| 54 of 59 | 17 | 372-KKKKADETQALPQRQKK-388 |
| 55 of 59 | 17 | 379-TQALPQRQKKQQTVTLL-395 |
| 56 of 59 | 17 | 386-QKKQQTVTLLPAADLDD-402 |
| 57 of 59 | 17 | 393-TLLPAADLDDFSKQLQQ-409 |
| 58 of 59 | 17 | 400-LDDFSKQLQQSMSSADS-416 |
| 59 of 59 | 13 | 407-LQQSMSSADSTQA-419 |

This 59-peptide array spans the nucleocapsid (N) protein of the USA-WA1/2020 strain of SARS-CoV-2 (GenPept. QH060601).
