# Supplementary material for: CHARM: COVID-19 Health Action Response for Marines–Association of antigen-specific interferon-gamma and IL2 responses with asymptomatic and symptomatic infections after a positive qPCR SARS-CoV-2 test
Source: PLoS One. 2022 Apr 7;17(4):e0266691. doi: 10.1371/journal.pone.0266691 (PMC8989306; doi:10.1371/journal.pone.0266691)
Supplement: S3 Table — This 31-peptide array spans the membrane (M) glycoprotein of the USA-WA1/2020 strain of SARS-CoV-2 (GenPept. QH060597). (DOCX) [file pone.0266691.s005.docx]

**Table S3. M protein peptides**

| Peptide | Length | Sequence |
| --- | --- | --- |
| 1 of 31 | 17 | 1-MADSNGTITVEELKKLL-17 |
| 2 of 31 | 17 | 8-ITVEELKKLLEQWNLVI-24 |
| 3 of 31 | 17 | 15-KLLEQWNLVIGFLFLTW-31 |
| 4 of 31 | 17 | 22-LVIGFLFLTWICLLQFA-38 |
| 5 of 31 | 17 | 29-LTWICLLQFAYANRNRF-45 |
| 6 of 31 | 17 | 36-QFAYANRNRFLYIIKLI-52 |
| 7 of 31 | 17 | 43-NRFLYIIKLIFLWLLWP-59 |
| 8 of 31 | 17 | 50-KLIFLWLLWPVTLACFV-66 |
| 9 of 31 | 17 | 57-LWPVTLACFVLAAVYRI-73 |
| 10 of 31 | 17 | 64-CFVLAAVYRINWITGGI-80 |
| 11 of 31 | 17 | 71-YRINWITGGIAIAMACL-87 |
| 12 of 31 | 17 | 78-GGIAIAMACLVGLMWLS-94 |
| 13 of 31 | 17 | 85-ACLVGLMWLSYFIASFR-101 |
| 14 of 31 | 17 | 92-WLSYFIASFRLFARTRS-108 |
| 15 of 31 | 17 | 99-SFRLFARTRSMWSFNPE-115 |
| 16 of 31 | 17 | 106-TRSMWSFNPETNILLNV-122 |
| 17 of 31 | 17 | 113-NPETNILLNVPLHGTIL-129 |
| 18 of 31 | 17 | 120-LNVPLHGTILTRPLLES-136 |
| 19 of 31 | 17 | 127-TILTRPLLESELVIGAV-143 |
| 20 of 31 | 17 | 134-LESELVIGAVILRGHLR-150 |
| 21 of 31 | 17 | 141-GAVILRGHLRIAGHHLG-157 |
| 22 of 31 | 17 | 148-HLRIAGHHLGRCDIKDL-164 |
| 23 of 31 | 17 | 155-HLGRCDIKDLPKEITVA-171 |
| 24 of 31 | 17 | 162-KDLPKEITVATSRTLSY-178 |
| 25 of 31 | 17 | 169-TVATSRTLSYYKLGASQ-185 |
| 26 of 31 | 17 | 176-LSYYKLGASQRVAGDSG-192 |
| 27 of 31 | 17 | 183-ASQRVAGDSGFAAYSRY-199 |
| 28 of 31 | 17 | 190-DSGFAAYSRYRIGNYKL-206 |
| 29 of 31 | 17 | 197-SRYRIGNYKLNTDHSSS-213 |
| 30 of 31 | 17 | 204-YKLNTDHSSSSDNIALL-220 |
| 31 of 31 | 12 | 211-SSSSDNIALLVQ-222 |

This 31-peptide array spans the membrane (M) glycoprotein of the USA-WA1/2020 strain of SARS-CoV-2 (GenPept. QH060597).
