# Supplementary material for: CHARM: COVID-19 Health Action Response for Marines–Association of antigen-specific interferon-gamma and IL2 responses with asymptomatic and symptomatic infections after a positive qPCR SARS-CoV-2 test
Source: PLoS One. 2022 Apr 7;17(4):e0266691. doi: 10.1371/journal.pone.0266691 (PMC8989306; doi:10.1371/journal.pone.0266691)
Supplement: S4 Table — This 241-peptide array corresponds to 221 predicted HLA class II CD4+ T cell epitopes covering all proteins in the viral genome except the spike (S) glycoprotein. (DOCX) [file pone.0266691.s006.docx]

**Table S4. CD4+ peptides**

|  | **Protein ID** | **Protein name** | **Peptide start** | **Peptide end** | **Peptide** | **Length** |
| --- | --- | --- | --- | --- | --- | --- |
| 1 | YP_009724389.1 | nsp1 | 11 | 25 | KTHVQLSLPVLQVRD | 15 |
| 2 | YP_009724389.1 | nsp1 | 66 | 80 | QPYVFIKRSDARTAP | 15 |
| 3 | YP_009724389.1 | nsp2 | 271 | 285 | PNFVFPLNSIIKTIQ | 15 |
| 4 | YP_009724389.1 | nsp2 | 276 | 290 | PLNSIIKTIQPRVEK | 15 |
| 5 | YP_009724389.1 | nsp2 | 296 | 310 | FMGRIRSVYPVASPN | 15 |
| 6 | YP_009724389.1 | nsp2 | 461 | 475 | INIVGDFKLNEEIAI | 15 |
| 7 | YP_009724389.1 | nsp2 | 471 | 485 | EEIAIILASFSASTS | 15 |
| 8 | YP_009724389.1 | nsp2 | 476 | 490 | ILASFSASTSAFVET | 15 |
| 9 | YP_009724389.1 | nsp2 | 531 | 545 | SPLYAFASEAARVVR | 15 |
| 10 | YP_009724389.1 | nsp2 | 541 | 555 | ARVVRSIFSRTLETA | 15 |
| 11 | YP_009724389.1 | nsp2 | 556 | 570 | QNSVRVLQKAAITIL | 15 |
| 12 | YP_009724389.1 | nsp2 | 566 | 580 | AITILDGISQYSLRL | 15 |
| 13 | YP_009724389.1 | nsp2 | 576 | 590 | YSLRLIDAMMFTSDL | 15 |
| 14 | YP_009724389.1 | nsp2 | 646 | 660 | WEIVKFISTCACEIV | 15 |
| 15 | YP_009724389.1 | nsp2 | 676 | 690 | QTFFKLVNKFLALCA | 15 |
| 16 | YP_009724389.1 | nsp2 | 706 | 720 | GETFVTHSKGLYRKC | 15 |
| 17 | YP_009724389.1 | PLpro | 1026 | 1040 | NSFSGYLKLTDNVYI | 15 |
| 18 | YP_009724389.1 | PLpro | 1051 | 1065 | KVKPTVVVNAANVYL | 15 |
| 19 | YP_009724389.1 | PLpro | 1151 | 1165 | AGIFGADPIHSLRVC | 15 |
| 20 | YP_009724389.1 | PLpro | 1251 | 1265 | ENLLLYIDINGNLHP | 15 |
| 21 | YP_009724389.1 | PLpro | 1276 | 1290 | ITFLKKDAPYIVGDV | 15 |
| 22 | YP_009724389.1 | PLpro | 1351 | 1365 | SAFYILPSIISNEKQ | 15 |
| 23 | YP_009724389.1 | PLpro | 1366 | 1380 | EILGTVSWNLREMLA | 15 |
| 24 | YP_009724389.1 | PLpro | 1421 | 1435 | RFYFYTSKTTVASLI | 15 |
| 25 | YP_009724389.1 | PLpro | 1461 | 1475 | EAARYMRSLKVPATV | 15 |
| 26 | YP_009724389.1 | PLpro | 1531 | 1545 | GDKSVYYTSNPTTFH | 15 |
| 27 | YP_009724389.1 | PLpro | 1556 | 1570 | LKTLLSLREVRTIKV | 15 |
| 28 | YP_009724389.1 | PLpro | 1801 | 1815 | ESPFVMMSAPPAQYE | 15 |
| 29 | YP_009724389.1 | PLpro | 1926 | 1940 | DNFKFVCDNIKFADD | 15 |
| 30 | YP_009724389.1 | PLpro | 1956 | 1970 | KVTFFPDLNGDVVAI | 15 |
| 31 | YP_009724389.1 | PLpro | 2111 | 2125 | NELSRVLGLKTLATH | 15 |
| 32 | YP_009724389.1 | PLpro | 2211 | 2225 | LEASFNYLKSPNFSK | 15 |
| 33 | YP_009724389.1 | PLpro | 2221 | 2235 | PNFSKLINIIIWFLL | 15 |
| 34 | YP_009724389.1 | PLpro | 2241 | 2255 | GSLIYSTAALGVLMS | 15 |
| 35 | YP_009724389.1 | PLpro | 2311 | 2325 | ISSFKWDLTAFGLVA | 15 |
| 36 | YP_009724389.1 | PLpro | 2326 | 2340 | EWFLAYILFTRFFYV | 15 |
| 37 | YP_009724389.1 | PLpro | 2336 | 2350 | RFFYVLGLAAIMQLF | 15 |
| 38 | YP_009724389.1 | PLpro | 2346 | 2360 | IMQLFFSYFAVHFIS | 15 |
| 39 | YP_009724389.1 | PLpro | 2351 | 2365 | FSYFAVHFISNSWLM | 15 |
| 40 | YP_009724389.1 | PLpro | 2361 | 2375 | NSWLMWLIINLVQMA | 15 |
| 41 | YP_009724389.1 | PLpro | 2366 | 2380 | WLIINLVQMAPISAM | 15 |
| 42 | YP_009724389.1 | PLpro | 2371 | 2385 | LVQMAPISAMVRMYI | 15 |
| 43 | YP_009724389.1 | PLpro | 2381 | 2395 | VRMYIFFASFYYVWK | 15 |
| 44 | YP_009724389.1 | PLpro | 2386 | 2400 | FFASFYYVWKSYVHV | 15 |
| 45 | YP_009724389.1 | PLpro | 2516 | 2530 | HSLSHFVNLDNLRAN | 15 |
| 46 | YP_009724389.1 | PLpro | 2566 | 2580 | CQPILLLDQALVSDV | 15 |
| 47 | YP_009724389.1 | PLpro | 2591 | 2605 | FDAYVNTFSSTFNVP | 15 |
| 48 | YP_009724389.1 | PLpro | 2621 | 2635 | AKNVSLDNVLSTFIS | 15 |
| 49 | YP_009724389.1 | PLpro | 2631 | 2645 | STFISAARQGFVDSD | 15 |
| 50 | YP_009724389.1 | PLpro | 2706 | 2720 | SHNIALIWNVKDFMS | 15 |
| 51 | YP_009724389.1 | nsp4 | 2761 | 2775 | KGGKIVNNWLKQLIK | 15 |
| 52 | YP_009724389.1 | nsp4 | 2776 | 2790 | VTLVFLFVAAIFYLI | 15 |
| 53 | YP_009724389.1 | nsp4 | 2781 | 2795 | LFVAAIFYLITPVHV | 15 |
| 54 | YP_009724389.1 | nsp4 | 2786 | 2800 | IFYLITPVHVMSKHT | 15 |
| 55 | YP_009724389.1 | nsp4 | 2871 | 2885 | GTILRTTNGDFLHFL | 15 |
| 56 | YP_009724389.1 | nsp4 | 2891 | 2905 | AVGNICYTPSKLIEY | 15 |
| 57 | YP_009724389.1 | nsp4 | 2951 | 2965 | DTRYVLMDGSIIQFP | 15 |
| 58 | YP_009724389.1 | nsp4 | 3001 | 3015 | SGRWVLNNDYYRSLP | 15 |
| 59 | YP_009724389.1 | nsp4 | 3006 | 3020 | LNNDYYRSLPGVFCG | 15 |
| 60 | YP_009724389.1 | nsp4 | 3016 | 3030 | GVFCGVDAVNLLTNM | 15 |
| 61 | YP_009724389.1 | nsp4 | 3061 | 3075 | AYYFMRFRRAFGEYS | 15 |
| 62 | YP_009724389.1 | nsp4 | 3071 | 3085 | FGEYSHVVAFNTLLF | 15 |
| 63 | YP_009724389.1 | nsp4 | 3081 | 3095 | NTLLFLMSFTVLCLT | 15 |
| 64 | YP_009724389.1 | nsp4 | 3086 | 3100 | LMSFTVLCLTPVYSF | 15 |
| 65 | YP_009724389.1 | nsp4 | 3111 | 3125 | YLTFYLTNDVSFLAH | 15 |
| 66 | YP_009724389.1 | nsp4 | 3121 | 3135 | SFLAHIQWMVMFTPL | 15 |
| 67 | YP_009724389.1 | nsp4 | 3126 | 3140 | IQWMVMFTPLVPFWI | 15 |
| 68 | YP_009724389.1 | nsp4 | 3136 | 3150 | VPFWITIAYIICIST | 15 |
| 69 | YP_009724389.1 | nsp4 | 3141 | 3155 | TIAYIICISTKHFYW | 15 |
| 70 | YP_009724389.1 | nsp4 | 3151 | 3165 | KHFYWFFSNYLKRRV | 15 |
| 71 | YP_009724389.1 | nsp4 | 3181 | 3195 | CTFLLNKEMYLKLRS | 15 |
| 72 | YP_009724389.1 | nsp4 | 3186 | 3200 | NKEMYLKLRSDVLLP | 15 |
| 73 | YP_009724389.1 | 3CL | 3316 | 3330 | NYEDLLIRKSNHNFL | 15 |
| 74 | YP_009724389.1 | 3CL | 3326 | 3340 | NHNFLVQAGNVQLRV | 15 |
| 75 | YP_009724389.1 | 3CL | 3346 | 3360 | QNCVLKLKVDTANPK | 15 |
| 76 | YP_009724389.1 | 3CL | 3361 | 3375 | TPKYKFVRIQPGQTF | 15 |
| 77 | YP_009724389.1 | 3CL | 3366 | 3380 | FVRIQPGQTFSVLAC | 15 |
| 78 | YP_009724389.1 | 3CL | 3471 | 3485 | LYAAVINGDRWFLNR | 15 |
| 79 | YP_009724389.1 | nsp6 | 3581 | 3595 | HWLLLTILTSLLVLV | 15 |
| 80 | YP_009724389.1 | nsp6 | 3591 | 3605 | LLVLVQSTQWSLFFF | 15 |
| 81 | YP_009724389.1 | nsp6 | 3601 | 3615 | SLFFFLYENAFLPFA | 15 |
| 82 | YP_009724389.1 | nsp6 | 3611 | 3625 | FLPFAMGIIAMSAFA | 15 |
| 83 | YP_009724389.1 | nsp6 | 3616 | 3630 | MGIIAMSAFAMMFVK | 15 |
| 84 | YP_009724389.1 | nsp6 | 3626 | 3640 | MMFVKHKHAFLCLFL | 15 |
| 85 | YP_009724389.1 | nsp6 | 3636 | 3650 | LCLFLLPSLATVAYF | 15 |
| 86 | YP_009724389.1 | nsp6 | 3651 | 3665 | NMVYMPASWVMRIMT | 15 |
| 87 | YP_009724389.1 | nsp6 | 3691 | 3705 | LLILMTARTVYDDGA | 15 |
| 88 | YP_009724389.1 | nsp6 | 3706 | 3720 | RRVWTLMNVLTLVYK | 15 |
| 89 | YP_009724389.1 | nsp6 | 3716 | 3730 | TLVYKVYYGNALDQA | 15 |
| 90 | YP_009724389.1 | nsp6 | 3731 | 3745 | ISMWALIISVTSNYS | 15 |
| 91 | YP_009724389.1 | nsp6 | 3736 | 3750 | LIISVTSNYSGVVTT | 15 |
| 92 | YP_009724389.1 | nsp6 | 3766 | 3780 | CPIFFITGNTLQCIM | 15 |
| 93 | YP_009724389.1 | nsp6 | 3801 | 3815 | NRYFRLTLGVYDYLV | 15 |
| 94 | YP_009724389.1 | nsp6 | 3811 | 3825 | YDYLVSTQEFRYMNS | 15 |
| 95 | YP_009724389.1 | nsp6 | 3816 | 3830 | STQEFRYMNSQGLLP | 15 |
| 96 | YP_009724389.1 | nsp6 | 3836 | 3850 | DAFKLNIKLLGVGGK | 15 |
| 97 | YP_009724389.1 | nsp7 | 3866 | 3880 | KCTSVVLLSVLQQLR | 15 |
| 98 | YP_009724389.1 | nsp7 | 3871 | 3885 | VLLSVLQQLRVESSS | 15 |
| 99 | YP_009724389.1 | nsp7 | 3876 | 3890 | LQQLRVESSSKLWAQ | 15 |
| 100 | YP_009724389.1 | nsp7 | 3906 | 3920 | EAFEKMVSLLSVLLS | 15 |
| 101 | YP_009724389.1 | nsp8 | 3946 | 3960 | SEFSSLPSYAAFATA | 15 |
| 102 | YP_009724389.1 | nsp8 | 3976 | 3990 | VLKKLKKSLNVAKSE | 15 |
| 103 | YP_009724389.1 | nsp8 | 4056 | 4070 | CVPLNIIPLTTAAKL | 15 |
| 104 | YP_009724389.1 | nsp8 | 4061 | 4075 | IIPLTTAAKLMVVIP | 15 |
| 105 | YP_009724389.1 | nsp8 | 4126 | 4140 | LIVTALRANSAVKLQ | 15 |
| 106 | YP_009724389.1 | nsp9 | 4226 | 4240 | KYLYFIKGLNNLNRG | 15 |
| 107 | YP_009724389.1 | nsp9 | 4241 | 4255 | MVLGSLAATVRLQAG | 15 |
| 108 | YP_009724389.1 | nsp10 | 4266 | 4280 | VLSFCAFAVDAAKAY | 15 |
| 109 | YP_009724389.1 | nsp10 | 4271 | 4285 | AFAVDAAKAYKDYLA | 15 |
| 110 | YP_009724389.1 | RdRpol | 4561 | 4575 | PDILRVYANLGERVR | 15 |
| 111 | YP_009724389.1 | RdRpol | 4626 | 4640 | VDSYYSLLMPILTLT | 15 |
| 112 | YP_009724389.1 | RdRpol | 4631 | 4645 | SLLMPILTLTRALTA | 15 |
| 113 | YP_009724389.1 | RdRpol | 4721 | 4735 | LVRKIFVDGVPFVVS | 15 |
| 114 | YP_009724389.1 | RdRpol | 4726 | 4740 | FVDGVPFVVSTGYHF | 15 |
| 115 | YP_009724389.1 | RdRpol | 4731 | 4745 | PFVVSTGYHFRELGV | 15 |
| 116 | YP_009724389.1 | RdRpol | 4761 | 4775 | KELLVYAADPAMHAA | 15 |
| 117 | YP_009724389.1 | RdRpol | 4931 | 4945 | ITQMNLKYAISAKNR | 15 |
| 118 | YP_009724389.1 | RdRpol | 4961 | 4975 | RQFHQKLLKSIAATR | 15 |
| 119 | YP_009724389.1 | RdRpol | 4966 | 4980 | KLLKSIAATRGATVV | 15 |
| 120 | YP_009724389.1 | RdRpol | 5016 | 5030 | RAMPNMLRIMASLVL | 15 |
| 121 | YP_009724389.1 | RdRpol | 5021 | 5035 | MLRIMASLVLARKHT | 15 |
| 122 | YP_009724389.1 | RdRpol | 5096 | 5110 | VNALLSTDGNKIADK | 15 |
| 123 | YP_009724389.1 | RdRpol | 5106 | 5120 | KIADKYVRNLQHRLY | 15 |
| 124 | YP_009724389.1 | RdRpol | 5136 | 5150 | EFYAYLRKHFSMMIL | 15 |
| 125 | YP_009724389.1 | RdRpol | 5141 | 5155 | LRKHFSMMILSDDAV | 15 |
| 126 | YP_009724389.1 | RdRpol | 5166 | 5180 | GLVASIKNFKSVLYY | 15 |
| 127 | YP_009724389.1 | RdRpol | 5236 | 5250 | VDDIVKTDGTLMIER | 15 |
| 128 | YP_009724389.1 | RdRpol | 5246 | 5260 | LMIERFVSLAIDAYP | 15 |
| 129 | YP_009724389.1 | Hel | 5361 | 5375 | TSHKLVLSVNPYVCN | 15 |
| 130 | YP_009724389.1 | Hel | 5451 | 5465 | TERLKLFAAETLKAT | 15 |
| 131 | YP_009724389.1 | Hel | 5466 | 5480 | EETFKLSYGIATVRE | 15 |
| 132 | YP_009724389.1 | Hel | 5506 | 5520 | FTGYRVTKNSKVQIG | 15 |
| 133 | YP_009724389.1 | Hel | 5531 | 5545 | DAVVYRGTTTYKLNV | 15 |
| 134 | YP_009724389.1 | Hel | 5546 | 5560 | GDYFVLTSHTVMPLS | 15 |
| 135 | YP_009724389.1 | Hel | 5611 | 5625 | GKSHFAIGLALYYPS | 15 |
| 136 | YP_009724389.1 | Hel | 5616 | 5630 | AIGLALYYPSARIVY | 15 |
| 137 | YP_009724389.1 | Hel | 5676 | 5690 | LEQYVFCTVNALPET | 15 |
| 138 | YP_009724389.1 | Hel | 5706 | 5720 | YDLSVVNARLRAKHY | 15 |
| 139 | YP_009724389.1 | Hel | 5796 | 5810 | FKMFYKGVITHDVSS | 15 |
| 140 | YP_009724389.1 | Hel | 5801 | 5815 | KGVITHDVSSAINRP | 15 |
| 141 | YP_009724389.1 | Hel | 5836 | 5850 | ISPYNSQNAVASKIL | 15 |
| 142 | YP_009724389.1 | Hel | 5881 | 5895 | NVNRFNVAITRAKVG | 15 |
| 143 | YP_009724389.1 | nsp14 | 5971 | 5985 | PKDMTYRRLISMMGF | 15 |
| 144 | YP_009724389.1 | nsp14 | 5976 | 5990 | YRRLISMMGFKMNYQ | 15 |
| 145 | YP_009724389.1 | nsp14 | 5996 | 6010 | NMFITREEAIRHVRA | 15 |
| 146 | YP_009724389.1 | nsp14 | 6001 | 6015 | REEAIRHVRAWIGFD | 15 |
| 147 | YP_009724389.1 | nsp14 | 6066 | 6080 | PPGDQFKHLIPLMYK | 15 |
| 148 | YP_009724389.1 | nsp14 | 6071 | 6085 | FKHLIPLMYKGLPWN | 15 |
| 149 | YP_009724389.1 | nsp14 | 6076 | 6090 | PLMYKGLPWNVVRIK | 15 |
| 150 | YP_009724389.1 | nsp14 | 6081 | 6095 | GLPWNVVRIKIVQML | 15 |
| 151 | YP_009724389.1 | nsp14 | 6086 | 6100 | VVRIKIVQMLSDTLK | 15 |
| 152 | YP_009724389.1 | nsp14 | 6091 | 6105 | IVQMLSDTLKNLSDR | 15 |
| 153 | YP_009724389.1 | nsp14 | 6111 | 6125 | WAHGFELTSMKYFVK | 15 |
| 154 | YP_009724389.1 | nsp14 | 6156 | 6170 | IGFDYVYNPFMIDVQ | 15 |
| 155 | YP_009724389.1 | nsp14 | 6236 | 6250 | KVQHMVVKAALLADK | 15 |
| 156 | YP_009724389.1 | nsp14 | 6286 | 6300 | YKIEELFYSYATHSD | 15 |
| 157 | YP_009724389.1 | nsp14 | 6356 | 6370 | FDKSAFVNLKQLPFF | 15 |
| 158 | YP_009724389.1 | nsp14 | 6416 | 6430 | YRLYLDAYNMMISAG | 15 |
| 159 | YP_009724389.1 | nsp14 | 6421 | 6435 | DAYNMMISAGFSLWV | 15 |
| 160 | YP_009724389.1 | nsp14 | 6521 | 6535 | VKILNNLGVDIAANT | 15 |
| 161 | YP_009724389.1 | nsp14 | 6536 | 6550 | VIWDYKRDAPAHIST | 15 |
| 162 | YP_009724389.1 | nsp15 | 6581 | 6595 | GQVDLFRNARNGVLI | 15 |
| 163 | YP_009724389.1 | nsp15 | 6696 | 6710 | QLGGLHLLIGLAKRF | 15 |
| 164 | YP_009724389.1 | nsp15 | 6716 | 6730 | ELEDFIPMDSTVKNY | 15 |
| 165 | YP_009724389.1 | nsp15 | 6756 | 6770 | EIIKSQDLSVVSKVV | 15 |
| 166 | YP_009724389.1 | nsp16 | 6836 | 6850 | KGIMMNVAKYTQLCQ | 15 |
| 167 | YP_009724389.1 | nsp16 | 6846 | 6860 | TQLCQYLNTLTLAVP | 15 |
| 168 | YP_009724389.1 | nsp16 | 6851 | 6865 | YLNTLTLAVPYNMRV | 15 |
| 169 | YP_009724389.1 | nsp16 | 7016 | 7030 | QIDGYVMHANYIFWR | 15 |
| 170 | YP_009724389.1 | nsp16 | 7031 | 7045 | NTNPIQLSSYSLFDM | 15 |
| 171 | YP_009724389.1 | nsp16 | 7066 | 7080 | NDMILSLLSKGRLII | 15 |
| 172 | YP_009724389.1 | nsp16 | 7076 | 7090 | GRLIIRENNRVVISS | 15 |
| 173 | YP_009724389.1 | nsp16 | 7081 | 7095 | RENNRVVISSDVLVN | 15 |
| 174 | YP_009724389.1 | nsp16 | 7082 | 7096 | ENNRVVISSDVLVNN | 15 |
| 175 | YP_009724390.1 | surface glycoprotein | 51 | 65 | TQDLFLPFFSNVTWF | 15 |
| 176 | YP_009724390.1 | surface glycoprotein | 56 | 70 | LPFFSNVTWFHAIHV | 15 |
| 177 | YP_009724390.1 | surface glycoprotein | 116 | 130 | SLLIVNNATNVVIKV | 15 |
| 178 | YP_009724390.1 | surface glycoprotein | 156 | 170 | EFRVYSSANNCTFEY | 15 |
| 179 | YP_009724390.1 | surface glycoprotein | 191 | 205 | EFVFKNIDGYFKIYS | 15 |
| 180 | YP_009724390.1 | surface glycoprotein | 231 | 245 | IGINITRFQTLLALH | 15 |
| 181 | YP_009724390.1 | surface glycoprotein | 236 | 250 | TRFQTLLALHRSYLT | 15 |
| 182 | YP_009724390.1 | surface glycoprotein | 306 | 320 | FTVEKGIYQTSNFRV | 15 |
| 183 | YP_009724390.1 | surface glycoprotein | 316 | 330 | SNFRVQPTESIVRFP | 15 |
| 184 | YP_009724390.1 | surface glycoprotein | 341 | 355 | VFNATRFASVYAWNR | 15 |
| 185 | YP_009724390.1 | surface glycoprotein | 451 | 465 | YLYRLFRKSNLKPFE | 15 |
| 186 | YP_009724390.1 | surface glycoprotein | 461 | 475 | LKPFERDISTEIYQA | 15 |
| 187 | YP_009724390.1 | surface glycoprotein | 506 | 520 | QPYRVVVLSFELLHA | 15 |
| 188 | YP_009724390.1 | surface glycoprotein | 821 | 835 | LLFNKVTLADAGFIK | 15 |
| 189 | YP_009724390.1 | surface glycoprotein | 896 | 910 | IPFAMQMAYRFNGIG | 15 |
| 190 | YP_009724390.1 | surface glycoprotein | 911 | 925 | VTQNVLYENQKLIAN | 15 |
| 191 | YP_009724390.1 | surface glycoprotein | 971 | 985 | GAISSVLNDILSRLD | 15 |
| 192 | YP_009724390.1 | surface glycoprotein | 1016 | 1030 | AEIRASANLAATKMS | 15 |
| 193 | YP_009724390.1 | surface glycoprotein | 1061 | 1075 | VFLHVTYVPAQEKNF | 15 |
| 194 | YP_009724390.1 | surface glycoprotein | 1216 | 1230 | IWLGFIAGLIAIVMV | 15 |
| 195 | YP_009724391.1 | ORF3a protein | 1 | 15 | MDLFMRIFTIGTVTL | 15 |
| 196 | YP_009724391.1 | ORF3a protein | 26 | 40 | SDFVRATATIPIQAS | 15 |
| 197 | YP_009724391.1 | ORF3a protein | 51 | 65 | ALLAVFQSASKIITL | 15 |
| 198 | YP_009724391.1 | ORF3a protein | 66 | 80 | KKRWQLALSKGVHFV | 15 |
| 199 | YP_009724391.1 | ORF3a protein | 76 | 90 | GVHFVCNLLLLFVTV | 15 |
| 200 | YP_009724391.1 | ORF3a protein | 106 | 120 | LYLYALVYFLQSINF | 15 |
| 201 | YP_009724391.1 | ORF3a protein | 111 | 125 | LVYFLQSINFVRIIM | 15 |
| 202 | YP_009724391.1 | ORF3a protein | 116 | 130 | QSINFVRIIMRLWLC | 15 |
| 203 | YP_009724391.1 | ORF3a protein | 136 | 150 | KNPLLYDANYFLCWH | 15 |
| 204 | YP_009724391.1 | ORF3a protein | 206 | 220 | YFTSDYYQLYSTQLS | 15 |
| 205 | YP_009724392.1 | envelope protein | 6 | 20 | SEETGTLIVNSVLLF | 15 |
| 206 | YP_009724392.1 | envelope protein | 11 | 25 | TLIVNSVLLFLAFVV | 15 |
| 207 | YP_009724392.1 | envelope protein | 16 | 30 | SVLLFLAFVVFLLVT | 15 |
| 208 | YP_009724392.1 | envelope protein | 21 | 35 | LAFVVFLLVTLAILT | 15 |
| 209 | YP_009724392.1 | envelope protein | 26 | 40 | FLLVTLAILTALRLC | 15 |
| 210 | YP_009724392.1 | envelope protein | 31 | 45 | LAILTALRLCAYCCN | 15 |
| 211 | YP_009724392.1 | envelope protein | 51 | 65 | LVKPSFYVYSRVKNL | 15 |
| 212 | YP_009724392.1 | envelope protein | 56 | 70 | FYVYSRVKNLNSSRV | 15 |
| 213 | YP_009724393.1 | membrane glycoprotein | 31 | 45 | WICLLQFAYANRNRF | 15 |
| 214 | YP_009724393.1 | membrane glycoprotein | 41 | 55 | NRNRFLYIIKLIFLW | 15 |
| 215 | YP_009724393.1 | membrane glycoprotein | 61 | 75 | TLACFVLAAVYRINW | 15 |
| 216 | YP_009724393.1 | membrane glycoprotein | 91 | 105 | MWLSYFIASFRLFAR | 15 |
| 217 | YP_009724393.1 | membrane glycoprotein | 96 | 110 | FIASFRLFARTRSMW | 15 |
| 218 | YP_009724393.1 | membrane glycoprotein | 136 | 150 | SELVIGAVILRGHLR | 15 |
| 219 | YP_009724393.1 | membrane glycoprotein | 166 | 180 | KEITVATSRTLSYYK | 15 |
| 220 | YP_009724393.1 | membrane glycoprotein | 176 | 190 | LSYYKLGASQRVAGD | 15 |
| 221 | YP_009724394.1 | ORF6 protein | 1 | 15 | MFHLVDFQVTIAEIL | 15 |
| 222 | YP_009724394.1 | ORF6 protein | 11 | 25 | IAEILLIIMRTFKVS | 15 |
| 223 | YP_009724394.1 | ORF6 protein | 16 | 30 | LIIMRTFKVSIWNLD | 15 |
| 224 | YP_009724394.1 | ORF6 protein | 21 | 35 | TFKVSIWNLDYIINL | 15 |
| 225 | YP_009724394.1 | ORF6 protein | 26 | 40 | IWNLDYIINLIIKNL | 15 |
| 226 | YP_009724394.1 | ORF6 protein | 31 | 45 | YIINLIIKNLSKSLT | 15 |
| 227 | YP_009724395.1 | ORF7a protein | 1 | 15 | MKIILFLALITLATC | 15 |
| 228 | YP_009724395.1 | ORF7a protein | 71 | 85 | VKHVYQLRARSVSPK | 15 |
| 229 | YP_009724395.1 | ORF7a protein | 96 | 110 | LYSPIFLIVAAIVFI | 15 |
| 230 | YP_009724395.1 | ORF7a protein | 101 | 115 | FLIVAAIVFITLCFT | 15 |
| 231 | YP_009724396.1 | ORF8 protein | 6 | 20 | FLGIITTVAAFHQEC | 15 |
| 232 | YP_009724396.1 | ORF8 protein | 41 | 55 | FYSKWYIRVGARKSA | 15 |
| 233 | YP_009724396.1 | ORF8 protein | 46 | 60 | YIRVGARKSAPLIEL | 15 |
| 234 | YP_00972439 | nucleocapsid phosphoprotein | 81 | 95 | DDQIGYYRRATRRIR | 15 |
| 235 | YP_00972439 | nucleocapsid phosphoprotein | 216 | 230 | DAALALLLLDRLNQL | 15 |
| 236 | YP_00972439 | nucleocapsid phosphoprotein | 221 | 235 | LLLLDRLNQLESKMS | 15 |
| 237 | YP_00972439 | nucleocapsid phosphoprotein | 301 | 315 | WPQIAQFAPSASAFF | 15 |
| 238 | YP_00972439 | nucleocapsid phosphoprotein | 306 | 320 | QFAPSASAFFGMSRI | 15 |
| 239 | YP_00972439 | nucleocapsid phosphoprotein | 326 | 340 | PSGTWLTYTGAIKLD | 15 |
| 240 | YP_00972439 | nucleocapsid phosphoprotein | 346 | 360 | FKDQVILLNKHIDAY | 15 |
| 241 | YP_00972525 | ORF10 protein | 1 | 15 | MGYINVFAFPFTIYS | 15 |

This 241-peptide array corresponds to 221 predicted HLA class II CD4+ T cell epitopes covering all proteins in the viral genome except the spike (S) glycoprotein.
