# Supplementary material for: CHARM: COVID-19 Health Action Response for Marines–Association of antigen-specific interferon-gamma and IL2 responses with asymptomatic and symptomatic infections after a positive qPCR SARS-CoV-2 test
Source: PLoS One. 2022 Apr 7;17(4):e0266691. doi: 10.1371/journal.pone.0266691 (PMC8989306; doi:10.1371/journal.pone.0266691)
Supplement: S5 Table — This 628-peptide array spans the USA-WA1/2020 strain of SARS-CoV-2 (Grifoni et al 2020, reference 9). (DOCX) [file pone.0266691.s007.docx]

**Table S5. CD8+ peptides**

| **Protein ID** | **Protein name** | **Peptide start** | **Peptide end** | **Peptide** | **HLA class I allele restriction** | | | |
| --- | --- | --- | --- | --- | --- | --- | --- | --- |
| YP_009724389.1 | nsp1 | 3 | 11 | SLVPGFNEK | HLA-A*03:01 | HLA-A*11:01 |  |  |
| YP_009724389.1 | nsp1 | 52 | 60 | GLVEVEKGV | HLA-A*02:01 |  |  |  |
| YP_009724389.1 | nsp1 | 56 | 64 | VEKGVLPQL | HLA-B*40:01 |  |  |  |
| YP_009724389.1 | nsp1 | 77 | 85 | RTAPHGHVM | HLA-B*07:02 |  |  |  |
| YP_009724389.1 | nsp1 | 79 | 88 | APHGHVMVEL | HLA-B*07:02 |  |  |  |
| YP_009724389.1 | nsp1 | 84 | 92 | VMVELVAEL | HLA-A*02:01 |  |  |  |
| YP_009724389.1 | nsp1 | 90 | 97 | AELEGIQY | HLA-B*44:02 | HLA-B*44:03 |  |  |
| YP_009724389.1 | nsp1 | 103 | 111 | TLGVLVPHV | HLA-A*02:01 |  |  |  |
| YP_009724389.1 | nsp1 | 108 | 118 | VPHVGEIPVAY | HLA-B*35:01 | HLA-B*07:02 |  |  |
| YP_009724389.1 | nsp1 | 110 | 118 | HVGEIPVAY | HLA-B*35:01 |  |  |  |
| YP_009724389.1 | nsp1 | 112 | 123 | GEIPVAYRKVLL | HLA-B*40:01 | HLA-B*07:02 | HLA-B*08:01 |  |
| YP_009724389.1 | nsp1 | 135 | 143 | SYGADLKSF | HLA-A*23:01 | HLA-A*24:02 |  |  |
| YP_009724389.1 | nsp1 | 175 | 185 | RELNGGAYTRY | HLA-B*44:03 |  |  |  |
| YP_009724389.1 | nsp2 | 185 | 196 | YVDNNFCGPDGY | HLA-A*01:01 |  |  |  |
| YP_009724389.1 | nsp2 | 221 | 229 | FIDTKRGVY | HLA-A*01:01 |  |  |  |
| YP_009724389.1 | nsp2 | 232 | 241 | REHEHEIAWY | HLA-B*44:02 | HLA-B*44:03 |  |  |
| YP_009724389.1 | nsp2 | 241 | 249 | YTERSEKSY | HLA-A*01:01 |  |  |  |
| YP_009724389.1 | nsp2 | 249 | 259 | YELQTPFEIKL | HLA-B*40:01 |  |  |  |
| YP_009724389.1 | nsp2 | 255 | 263 | FEIKLAKKF | HLA-B*44:02 | HLA-B*44:03 |  |  |
| YP_009724389.1 | nsp2 | 282 | 291 | KTIQPRVEKK | HLA-A*03:01 | HLA-A*11:01 |  |  |
| YP_009724389.1 | nsp2 | 285 | 293 | QPRVEKKKL | HLA-B*07:02 |  |  |  |
| YP_009724389.1 | nsp2 | 372 | 384 | ACHNSEVGPEHSL | HLA-B*40:01 |  |  |  |
| YP_009724389.1 | nsp2 | 376 | 387 | SEVGPEHSLAEY | HLA-B*44:02 | HLA-B*40:01 | HLA-B*44:03 | HLA-B*35:01 |
| YP_009724389.1 | nsp2 | 385 | 393 | AEYHNESGL | HLA-B*40:01 |  |  |  |
| YP_009724389.1 | nsp2 | 389 | 397 | NESGLKTIL | HLA-B*40:01 |  |  |  |
| YP_009724389.1 | nsp2 | 443 | 451 | SEGLNDNLL | HLA-B*40:01 |  |  |  |
| YP_009724389.1 | nsp2 | 445 | 454 | GLNDNLLEIL | HLA-A*02:01 |  |  |  |
| YP_009724389.1 | nsp2 | 468 | 476 | KLNEEIAII | HLA-A*02:01 |  |  |  |
| YP_009724389.1 | nsp2 | 471 | 480 | EEIAIILASF | HLA-B*44:02 | HLA-B*44:03 |  |  |
| YP_009724389.1 | nsp2 | 483 | 492 | STSAFVETVK | HLA-A*11:01 |  |  |  |
| YP_009724389.1 | nsp2 | 510 | 518 | KVTKGKAKK | HLA-A*03:01 |  |  |  |
| YP_009724389.1 | nsp2 | 524 | 533 | GEQKSILSPL | HLA-B*40:01 |  |  |  |
| YP_009724389.1 | nsp2 | 542 | 550 | RVVRSIFSR | HLA-A*11:01 |  |  |  |
| YP_009724389.1 | nsp2 | 568 | 576 | TILDGISQY | HLA-B*35:01 |  |  |  |
| YP_009724389.1 | nsp2 | 579 | 587 | RLIDAMMFT | HLA-A*02:01 |  |  |  |
| YP_009724389.1 | nsp2 | 587 | 600 | TSDLATNNLVVMAY | HLA-A*01:01 |  |  |  |
| YP_009724389.1 | nsp2 | 600 | 608 | YITGGVVQL | HLA-A*02:01 |  |  |  |
| YP_009724389.1 | nsp2 | 615 | 623 | NIFGTVYEK | HLA-A*03:01 | HLA-A*11:01 | HLA-A*01:01 |  |
| YP_009724389.1 | nsp2 | 621 | 630 | YEKLKPVLDW | HLA-B*44:02 | HLA-B*44:03 |  |  |
| YP_009724389.1 | nsp2 | 632 | 641 | EEKFKEGVEF | HLA-B*44:02 | HLA-B*44:03 |  |  |
| YP_009724389.1 | nsp2 | 669 | 678 | KEIKESVQTF | HLA-B*40:01 | HLA-B*44:02 | HLA-B*44:03 |  |
| YP_009724389.1 | nsp2 | 677 | 685 | TFFKLVNKF | HLA-A*23:01 | HLA-A*24:02 | HLA-A*03:01 | HLA-A*11:01 |
| YP_009724389.1 | nsp2 | 680 | 688 | KLVNKFLAL | HLA-A*02:01 | HLA-B*08:01 |  |  |
| YP_009724389.1 | nsp2 | 693 | 701 | IIIGGAKLK | HLA-A*03:01 |  |  |  |
| YP_009724389.1 | nsp2 | 697 | 705 | GAKLKALNL | HLA-B*08:01 |  |  |  |
| YP_009724389.1 | nsp2 | 702 | 710 | ALNLGETFV | HLA-A*02:01 |  |  |  |
| YP_009724389.1 | nsp2 | 731 | 739 | MPLKAPKEI | HLA-B*07:02 |  |  |  |
| YP_009724389.1 | nsp2 | 735 | 742 | APKEIIFL | HLA-B*07:02 |  |  |  |
| YP_009724389.1 | nsp2 | 737 | 747 | KEIIFLEGETL | HLA-B*40:01 |  |  |  |
| YP_009724389.1 | nsp2 | 744 | 752 | GETLPTEVL | HLA-B*40:01 |  |  |  |
| YP_009724389.1 | nsp2 | 791 | 799 | LEIKDTEKY | HLA-B*44:02 | HLA-B*44:03 |  |  |
| YP_009724389.1 | nsp2 | 808 | 816 | VTNNTFTLK | HLA-A*03:01 | HLA-A*11:01 |  |  |
| YP_009724389.1 | nsp2 | 814 | 822 | TLKGGAPTK | HLA-A*03:01 |  |  |  |
| YP_009724389.1 | PLpro | 829 | 837 | TVIEVQGYK | HLA-A*03:01 | HLA-A*11:01 |  |  |
| YP_009724389.1 | PLpro | 831 | 839 | IEVQGYKSV | HLA-B*40:01 |  |  |  |
| YP_009724389.1 | PLpro | 834 | 843 | QGYKSVNITF | HLA-A*23:01 | HLA-A*24:02 |  |  |
| YP_009724389.1 | PLpro | 843 | 853 | FELDERIDKVL | HLA-B*40:01 | HLA-A*02:01 |  |  |
| YP_009724389.1 | PLpro | 848 | 856 | RIDKVLNEK | HLA-A*03:01 | HLA-A*11:01 |  |  |
| YP_009724389.1 | PLpro | 887 | 895 | SELLTPLGI | HLA-B*40:01 |  |  |  |
| YP_009724389.1 | PLpro | 898 | 906 | DEWSMATYY | HLA-B*44:02 | HLA-B*44:03 |  |  |
| YP_009724389.1 | PLpro | 906 | 916 | YLFDESGEFKL | HLA-A*02:01 | HLA-A*23:01 | HLA-A*24:02 |  |
| YP_009724389.1 | PLpro | 912 | 921 | GEFKLASHMY | HLA-B*44:02 | HLA-B*40:01 | HLA-B*44:03 |  |
| YP_009724389.1 | PLpro | 934 | 947 | GDCEEEEFEPSTQY | HLA-B*44:02 | HLA-B*44:03 |  |  |
| YP_009724389.1 | PLpro | 938 | 949 | EEEFEPSTQYEY | HLA-B*44:02 | HLA-B*44:03 |  |  |
| YP_009724389.1 | PLpro | 954 | 962 | DYQGKPLEF | HLA-A*23:01 | HLA-A*24:02 |  |  |
| YP_009724389.1 | PLpro | 958 | 969 | KPLEFGATSAAL | HLA-B*07:02 | HLA-B*40:01 |  |  |
| YP_009724389.1 | PLpro | 994 | 1002 | SEDNQTTTI | HLA-B*40:01 |  |  |  |
| YP_009724389.1 | PLpro | 1006 | 1014 | VEVQPQLEM | HLA-B*40:01 | HLA-B*44:03 |  |  |
| YP_009724389.1 | PLpro | 1012 | 1023 | LEMELTPVVQTI | HLA-B*40:01 |  |  |  |
| YP_009724389.1 | PLpro | 1023 | 1031 | IEVNSFSGY | HLA-B*44:02 | HLA-B*44:03 |  |  |
| YP_009724389.1 | PLpro | 1031 | 1041 | YLKLTDNVYIK | HLA-A*02:01 | HLA-A*03:01 |  |  |
| YP_009724389.1 | PLpro | 1057 | 1066 | VVNAANVYLK | HLA-A*11:01 |  |  |  |
| YP_009724389.1 | PLpro | 1104 | 1112 | VLSGHNLAK | HLA-A*03:01 |  |  |  |
| YP_009724389.1 | PLpro | 1116 | 1124 | HVVGPNVNK | HLA-A*03:01 | HLA-A*11:01 |  |  |
| YP_009724389.1 | PLpro | 1135 | 1144 | YENFNQHEVL | HLA-B*40:01 |  |  |  |
| YP_009724389.1 | PLpro | 1141 | 1149 | HEVLLAPLL | HLA-B*40:01 |  |  |  |
| YP_009724389.1 | PLpro | 1146 | 1154 | APLLSAGIF | HLA-B*07:02 |  |  |  |
| YP_009724389.1 | PLpro | 1176 | 1185 | AVFDKNLYDK | HLA-A*03:01 | HLA-A*11:01 |  |  |
| YP_009724389.1 | PLpro | 1182 | 1190 | LYDKLVSSF | HLA-A*23:01 | HLA-A*24:02 |  |  |
| YP_009724389.1 | PLpro | 1195 | 1203 | SEKQVEQKI | HLA-B*44:02 | HLA-B*44:03 |  |  |
| YP_009724389.1 | PLpro | 1204 | 1214 | AEIPKEEVKPF | HLA-B*44:02 | HLA-B*07:02 | HLA-B*35:01 |  |
| YP_009724389.1 | PLpro | 1249 | 1256 | LTENLLLY | HLA-A*01:01 |  |  |  |
| YP_009724389.1 | PLpro | 1255 | 1263 | LYIDINGNL | HLA-A*23:01 |  |  |  |
| YP_009724389.1 | PLpro | 1270 | 1280 | LVSDIDITFLK | HLA-A*11:01 | HLA-B*35:01 |  |  |
| YP_009724389.1 | PLpro | 1278 | 1286 | FLKKDAPYI | HLA-A*02:01 |  |  |  |
| YP_009724389.1 | PLpro | 1283 | 1291 | APYIVGDVV | HLA-B*07:02 |  |  |  |
| YP_009724389.1 | PLpro | 1296 | 1304 | LTAVVIPTK | HLA-A*11:01 |  |  |  |
| YP_009724389.1 | PLpro | 1316 | 1329 | ALRKVPTDNYITTY | HLA-A*01:01 |  |  |  |
| YP_009724389.1 | PLpro | 1319 | 1329 | KVPTDNYITTY | HLA-A*01:01 | HLA-B*35:01 |  |  |
| YP_009724389.1 | PLpro | 1329 | 1337 | YPGQGLNGY | HLA-B*35:01 |  |  |  |
| YP_009724389.1 | PLpro | 1361 | 1373 | SNEKQEILGTVSW | HLA-B*44:02 | HLA-B*44:03 |  |  |
| YP_009724389.1 | PLpro | 1387 | 1396 | KLMPVCVETK | HLA-A*03:01 |  |  |  |
| YP_009724389.1 | PLpro | 1397 | 1405 | AIVSTIQRK | HLA-A*03:01 | HLA-A*11:01 |  |  |
| YP_009724389.1 | PLpro | 1412 | 1422 | QEGVVDYGARF | HLA-B*44:02 | HLA-B*44:03 |  |  |
| YP_009724389.1 | PLpro | 1415 | 1423 | VVDYGARFY | HLA-A*01:01 |  |  |  |
| YP_009724389.1 | PLpro | 1437 | 1445 | TLNDLNETL | HLA-A*02:01 |  |  |  |
| YP_009724389.1 | PLpro | 1442 | 1450 | NETLVTMPL | HLA-B*40:01 |  |  |  |
| YP_009724389.1 | PLpro | 1479 | 1489 | SPDAVTAYNGY | HLA-B*35:01 |  |  |  |
| YP_009724389.1 | PLpro | 1504 | 1512 | TISLAGSYK | HLA-A*03:01 | HLA-A*11:01 |  |  |
| YP_009724389.1 | PLpro | 1520 | 1529 | STQLGIEFLK | HLA-A*11:01 |  |  |  |
| YP_009724389.1 | PLpro | 1535 | 1544 | VYYTSNPTTF | HLA-A*23:01 | HLA-A*24:02 |  |  |
| YP_009724389.1 | PLpro | 1548 | 1556 | GEVITFDNL | HLA-B*40:01 |  |  |  |
| YP_009724389.1 | PLpro | 1555 | 1562 | NLKTLLSL | HLA-B*08:01 |  |  |  |
| YP_009724389.1 | PLpro | 1561 | 1571 | SLREVRTIKVF | HLA-B*40:01 | HLA-B*44:02 | HLA-B*44:03 | HLA-A*03:01 |
| YP_009724389.1 | PLpro | 1582 | 1590 | QVVDMSMTY | HLA-B*35:01 |  |  |  |
| YP_009724389.1 | PLpro | 1598 | 1607 | YLDGADVTKI | HLA-A*02:01 |  |  |  |
| YP_009724389.1 | PLpro | 1613 | 1621 | HEGKTFYVL | HLA-B*40:01 |  |  |  |
| YP_009724389.1 | PLpro | 1621 | 1634 | LPNDDTLRVEAFEY | HLA-B*35:01 |  |  |  |
| YP_009724389.1 | PLpro | 1633 | 1642 | EYYHTTDPSF | HLA-A*23:01 | HLA-A*24:02 |  |  |
| YP_009724389.1 | PLpro | 1636 | 1646 | HTTDPSFLGRY | HLA-A*01:01 |  |  |  |
| YP_009724389.1 | PLpro | 1658 | 1667 | YPQVNGLTSI | HLA-B*07:02 |  |  |  |
| YP_009724389.1 | PLpro | 1675 | 1683 | YLATALLTL | HLA-A*02:01 |  |  |  |
| YP_009724389.1 | PLpro | 1686 | 1695 | IELKFNPPAL | HLA-B*40:01 | HLA-B*08:01 |  |  |
| YP_009724389.1 | PLpro | 1691 | 1699 | NPPALQDAY | HLA-B*35:01 |  |  |  |
| YP_009724389.1 | PLpro | 1699 | 1710 | YYRARAGEAANF | HLA-A*24:02 |  |  |  |
| YP_009724389.1 | PLpro | 1705 | 1713 | GEAANFCAL | HLA-B*40:01 |  |  |  |
| YP_009724389.1 | PLpro | 1723 | 1734 | GELGDVRETMSY | HLA-B*44:02 | HLA-B*40:01 | HLA-B*44:03 |  |
| YP_009724389.1 | PLpro | 1733 | 1741 | SYLFQHANL | HLA-A*23:01 | HLA-A*24:02 |  |  |
| YP_009724389.1 | PLpro | 1767 | 1776 | AVMYMGTLSY | HLA-A*03:01 |  |  |  |
| YP_009724389.1 | PLpro | 1769 | 1779 | MYMGTLSYEQF | HLA-A*24:02 |  |  |  |
| YP_009724389.1 | PLpro | 1772 | 1780 | GTLSYEQFK | HLA-A*11:01 |  |  |  |
| YP_009724389.1 | PLpro | 1776 | 1785 | YEQFKKGVQI | HLA-B*40:01 |  |  |  |
| YP_009724389.1 | PLpro | 1825 | 1836 | SEYTGNYQCGHY | HLA-B*44:02 | HLA-B*44:03 |  |  |
| YP_009724389.1 | PLpro | 1857 | 1868 | SEYKGPITDVFY | HLA-B*40:01 | HLA-B*44:02 | HLA-B*44:03 | HLA-A*23:01 |
| YP_009724389.1 | PLpro | 1863 | 1873 | ITDVFYKENSY | HLA-A*01:01 |  |  |  |
| YP_009724389.1 | PLpro | 1869 | 1877 | KENSYTTTI | HLA-B*40:01 | HLA-B*44:02 | HLA-B*44:03 |  |
| YP_009724389.1 | PLpro | 1872 | 1883 | SYTTTIKPVTYK | HLA-A*11:01 | HLA-A*03:01 |  |  |
| YP_009724389.1 | PLpro | 1876 | 1884 | TIKPVTYKL | HLA-B*08:01 |  |  |  |
| YP_009724389.1 | PLpro | 1890 | 1900 | TEIDPKLDNYY | HLA-B*44:02 | HLA-B*44:03 | HLA-A*01:01 |  |
| YP_009724389.1 | PLpro | 1898 | 1907 | NYYKKDNSYF | HLA-A*23:01 | HLA-A*24:02 |  |  |
| YP_009724389.1 | PLpro | 1907 | 1920 | FTEQPIDLVPNQPY | HLA-A*01:01 |  |  |  |
| YP_009724389.1 | PLpro | 1919 | 1930 | PYPNASFDNFKF | HLA-A*24:02 | HLA-B*07:02 | HLA-B*35:01 |  |
| YP_009724389.1 | PLpro | 1937 | 1947 | FADDLNQLTGY | HLA-A*01:01 |  |  |  |
| YP_009724389.1 | PLpro | 1949 | 1959 | KPASRELKVTF | HLA-B*07:02 |  |  |  |
| YP_009724389.1 | PLpro | 1971 | 1979 | DYKHYTPSF | HLA-A*23:01 | HLA-A*24:02 |  |  |
| YP_009724389.1 | PLpro | 1976 | 1985 | TPSFKKGAKL | HLA-B*07:02 |  |  |  |
| YP_009724389.1 | PLpro | 2002 | 2010 | TYKPNTWCI | HLA-A*24:02 |  |  |  |
| YP_009724389.1 | PLpro | 2016 | 2025 | TKPVETSNSF | HLA-B*35:01 | HLA-B*07:02 |  |  |
| YP_009724389.1 | PLpro | 2021 | 2029 | TSNSFDVLK | HLA-A*11:01 |  |  |  |
| YP_009724389.1 | PLpro | 2051 | 2059 | VVENPTIQK | HLA-A*03:01 | HLA-A*11:01 |  |  |
| YP_009724389.1 | PLpro | 2069 | 2077 | TEVVGDIIL | HLA-B*40:01 |  |  |  |
| YP_009724389.1 | PLpro | 2076 | 2084 | ILKPANNSL | HLA-B*08:01 |  |  |  |
| YP_009724389.1 | PLpro | 2078 | 2086 | KPANNSLKI | HLA-B*07:02 |  |  |  |
| YP_009724389.1 | PLpro | 2086 | 2099 | ITEEVGHTDLMAAY | HLA-A*01:01 |  |  |  |
| YP_009724389.1 | PLpro | 2106 | 2113 | TIKKPNEL | HLA-B*08:01 |  |  |  |
| YP_009724389.1 | PLpro | 2108 | 2117 | KKPNELSRVL | HLA-B*07:02 |  |  |  |
| YP_009724389.1 | PLpro | 2111 | 2119 | NELSRVLGL | HLA-B*40:01 | HLA-B*44:03 |  |  |
| YP_009724389.1 | PLpro | 2133 | 2141 | VPWDTIANY | HLA-B*35:01 |  |  |  |
| YP_009724389.1 | PLpro | 2167 | 2175 | NYMPYFFTL | HLA-A*23:01 | HLA-A*24:02 |  |  |
| YP_009724389.1 | PLpro | 2169 | 2177 | MPYFFTLLL | HLA-B*07:02 |  |  |  |
| YP_009724389.1 | PLpro | 2187 | 2194 | NSRIKASM | HLA-B*08:01 |  |  |  |
| YP_009724389.1 | PLpro | 2191 | 2200 | KASMPTTIAK | HLA-A*03:01 | HLA-A*11:01 |  |  |
| YP_009724389.1 | PLpro | 2196 | 2204 | TTIAKNTVK | HLA-A*11:01 |  |  |  |
| YP_009724389.1 | PLpro | 2213 | 2225 | ASFNYLKSPNFSK | HLA-A*11:01 | HLA-A*03:01 |  |  |
| YP_009724389.1 | PLpro | 2270 | 2278 | YLNSTNVTI | HLA-A*02:01 |  |  |  |
| YP_009724389.1 | PLpro | 2301 | 2309 | YPSLETIQI | HLA-B*35:01 |  |  |  |
| YP_009724389.1 | PLpro | 2325 | 2334 | AEWFLAYILF | HLA-B*40:01 | HLA-A*23:01 |  |  |
| YP_009724389.1 | PLpro | 2330 | 2340 | AYILFTRFFYV | HLA-A*23:01 | HLA-A*24:02 | HLA-A*02:01 |  |
| YP_009724389.1 | PLpro | 2373 | 2383 | QMAPISAMVRM | HLA-A*02:01 | HLA-B*07:02 | HLA-B*35:01 |  |
| YP_009724389.1 | PLpro | 2386 | 2394 | FFASFYYVW | HLA-A*23:01 | HLA-A*24:02 |  |  |
| YP_009724389.1 | PLpro | 2392 | 2400 | YVWKSYVHV | HLA-A*02:01 |  |  |  |
| YP_009724389.1 | PLpro | 2436 | 2444 | VYANGGKGF | HLA-A*23:01 | HLA-A*24:02 |  |  |
| YP_009724389.1 | PLpro | 2469 | 2477 | VARDLSLQF | HLA-B*35:01 |  |  |  |
| YP_009724389.1 | PLpro | 2511 | 2518 | KTYERHSL | HLA-B*08:01 |  |  |  |
| YP_009724389.1 | PLpro | 2553 | 2561 | SAKSASVYY | HLA-B*35:01 |  |  |  |
| YP_009724389.1 | PLpro | 2569 | 2577 | ILLLDQALV | HLA-A*02:01 |  |  |  |
| YP_009724389.1 | PLpro | 2584 | 2594 | AEVAVKMFDAY | HLA-B*44:02 | HLA-B*44:03 | HLA-B*35:01 |  |
| YP_009724389.1 | PLpro | 2589 | 2598 | KMFDAYVNTF | HLA-A*23:01 | HLA-A*24:02 |  |  |
| YP_009724389.1 | PLpro | 2593 | 2602 | AYVNTFSSTF | HLA-A*23:01 | HLA-A*24:02 | HLA-B*35:01 |  |
| YP_009724389.1 | PLpro | 2599 | 2610 | SSTFNVPMEKLK | HLA-A*11:01 | HLA-A*03:01 |  |  |
| YP_009724389.1 | PLpro | 2603 | 2612 | NVPMEKLKTL | HLA-B*08:01 | HLA-B*07:02 |  |  |
| YP_009724389.1 | PLpro | 2614 | 2622 | ATAEAELAK | HLA-A*03:01 | HLA-A*11:01 |  |  |
| YP_009724389.1 | PLpro | 2616 | 2626 | AEAELAKNVSL | HLA-B*40:01 | HLA-B*44:02 | HLA-B*44:03 |  |
| YP_009724389.1 | PLpro | 2618 | 2630 | AELAKNVSLDNVL | HLA-B*40:01 | HLA-B*44:02 | HLA-B*44:03 | HLA-B*08:01 |
| YP_009724389.1 | PLpro | 2680 | 2688 | VENMTPRDL | HLA-B*40:01 |  |  |  |
| YP_009724389.1 | PLpro | 2684 | 2692 | TPRDLGACI | HLA-B*07:02 |  |  |  |
| YP_009724389.1 | PLpro | 2703 | 2711 | VAKSHNIAL | HLA-B*07:02 | HLA-B*08:01 |  |  |
| YP_009724389.1 | PLpro | 2714 | 2721 | NVKDFMSL | HLA-B*08:01 |  |  |  |
| YP_009724389.1 | PLpro | 2731 | 2738 | SAAKKNNL | HLA-B*08:01 |  |  |  |
| YP_009724389.1 | PLpro | 2748 | 2757 | RQVVNVVTTK | HLA-A*03:01 | HLA-A*11:01 |  |  |
| YP_009724389.1 | PLpro | 2753 | 2761 | VVTTKIALK | HLA-A*03:01 | HLA-B*08:01 |  |  |
| YP_009724389.1 | nsp4 | 2779 | 2787 | VFLFVAAIF | HLA-A*23:01 |  |  |  |
| YP_009724389.1 | nsp4 | 2788 | 2796 | YLITPVHVM | HLA-A*02:01 |  |  |  |
| YP_009724389.1 | nsp4 | 2794 | 2802 | HVMSKHTDF | HLA-B*08:01 |  |  |  |
| YP_009724389.1 | nsp4 | 2799 | 2809 | HTDFSSEIIGY | HLA-A*01:01 |  |  |  |
| YP_009724389.1 | nsp4 | 2802 | 2812 | FSSEIIGYKAI | HLA-B*40:01 | HLA-B*44:02 | HLA-B*44:03 | HLA-A*11:01 |
| YP_009724389.1 | nsp4 | 2860 | 2869 | REVGFVVPGL | HLA-B*40:01 |  |  |  |
| YP_009724389.1 | nsp4 | 2865 | 2874 | VVPGLPGTIL | HLA-B*07:02 |  |  |  |
| YP_009724389.1 | nsp4 | 2884 | 2892 | FLPRVFSAV | HLA-A*02:01 |  |  |  |
| YP_009724389.1 | nsp4 | 2889 | 2897 | FSAVGNICY | HLA-A*01:01 |  |  |  |
| YP_009724389.1 | nsp4 | 2901 | 2909 | KLIEYTDFA | HLA-A*02:01 |  |  |  |
| YP_009724389.1 | nsp4 | 2920 | 2928 | TIFKDASGK | HLA-A*03:01 |  |  |  |
| YP_009724389.1 | nsp4 | 2924 | 2932 | DASGKPVPY | HLA-B*35:01 |  |  |  |
| YP_009724389.1 | nsp4 | 2930 | 2939 | VPYCYDTNVL | HLA-B*07:02 |  |  |  |
| YP_009724389.1 | nsp4 | 2937 | 2945 | NVLEGSVAY | HLA-B*35:01 |  |  |  |
| YP_009724389.1 | nsp4 | 2949 | 2957 | RPDTRYVLM | HLA-B*07:02 | HLA-B*08:01 |  |  |
| YP_009724389.1 | nsp4 | 2960 | 2968 | SIIQFPNTY | HLA-B*35:01 |  |  |  |
| YP_009724389.1 | nsp4 | 2967 | 2979 | TYLEGSVRVVTTF | HLA-A*23:01 | HLA-A*24:02 |  |  |
| YP_009724389.1 | nsp4 | 2992 | 3004 | SEAGVCVSTSGRW | HLA-B*44:02 |  |  |  |
| YP_009724389.1 | nsp4 | 3010 | 3018 | YYRSLPGVF | HLA-A*23:01 | HLA-A*24:02 |  |  |
| YP_009724389.1 | nsp4 | 3032 | 3041 | TPLIQPIGAL | HLA-B*07:02 |  |  |  |
| YP_009724389.1 | nsp4 | 3047 | 3055 | IVAGGIVAI | HLA-A*02:01 |  |  |  |
| YP_009724389.1 | nsp4 | 3064 | 3071 | FMRFRRAF | HLA-B*08:01 |  |  |  |
| YP_009724389.1 | nsp4 | 3072 | 3084 | GEYSHVVAFNTLL | HLA-B*40:01 | HLA-B*44:02 | HLA-B*44:03 |  |
| YP_009724389.1 | nsp4 | 3100 | 3109 | FLPGVYSVIY | HLA-B*35:01 | HLA-A*02:01 |  |  |
| YP_009724389.1 | nsp4 | 3103 | 3112 | GVYSVIYLYL | HLA-A*23:01 | HLA-A*24:02 | HLA-A*03:01 | HLA-A*11:01 |
| YP_009724389.1 | nsp4 | 3113 | 3123 | TFYLTNDVSFL | HLA-A*23:01 | HLA-A*24:02 | HLA-A*02:01 |  |
| YP_009724389.1 | nsp4 | 3122 | 3130 | FLAHIQWMV | HLA-A*02:01 |  |  |  |
| YP_009724389.1 | nsp4 | 3131 | 3139 | MFTPLVPFW | HLA-A*23:01 | HLA-A*24:02 |  |  |
| YP_009724389.1 | nsp4 | 3136 | 3144 | VPFWITIAY | HLA-B*35:01 |  |  |  |
| YP_009724389.1 | nsp4 | 3159 | 3167 | NYLKRRVVF | HLA-A*24:02 | HLA-A*23:01 | HLA-B*08:01 |  |
| YP_009724389.1 | nsp4 | 3166 | 3175 | VFNGVSFSTF | HLA-A*23:01 | HLA-A*24:02 |  |  |
| YP_009724389.1 | nsp4 | 3179 | 3187 | ALCTFLLNK | HLA-A*03:01 |  |  |  |
| YP_009724389.1 | nsp4 | 3183 | 3191 | FLLNKEMYL | HLA-A*02:01 | HLA-B*08:01 |  |  |
| YP_009724389.1 | nsp4 | 3190 | 3198 | YLKLRSDVL | HLA-B*08:01 |  |  |  |
| YP_009724389.1 | nsp4 | 3199 | 3207 | LPLTQYNRY | HLA-B*35:01 |  |  |  |
| YP_009724389.1 | nsp4 | 3202 | 3210 | TQYNRYLAL | HLA-B*08:01 |  |  |  |
| YP_009724389.1 | nsp4 | 3219 | 3227 | GAMDTTSYR | HLA-A*11:01 |  |  |  |
| YP_009724389.1 | nsp4 | 3241 | 3250 | FSNSGSDVLY | HLA-A*01:01 |  |  |  |
| YP_009724389.1 | nsp4 | 3249 | 3257 | LYQPPQTSI | HLA-A*23:01 | HLA-A*24:02 |  |  |
| YP_009724389.1 | nsp4 | 3259 | 3268 | SAVLQSGFRK | HLA-A*11:01 | HLA-A*03:01 |  |  |
| YP_009724389.1 | 3CL | 3308 | 3317 | TSEDMLNPNY | HLA-A*01:01 | HLA-B*44:02 | HLA-B*44:03 |  |
| YP_009724389.1 | 3CL | 3329 | 3338 | FLVQAGNVQL | HLA-A*02:01 |  |  |  |
| YP_009724389.1 | 3CL | 3337 | 3345 | QLRVIGHSM | HLA-B*08:01 |  |  |  |
| YP_009724389.1 | 3CL | 3356 | 3364 | TANPKTPKY | HLA-B*35:01 |  |  |  |
| YP_009724389.1 | 3CL | 3361 | 3369 | TPKYKFVRI | HLA-B*08:01 |  |  |  |
| YP_009724389.1 | 3CL | 3363 | 3375 | KYKFVRIQPGQTF | HLA-A*23:01 | HLA-A*24:02 |  |  |
| YP_009724389.1 | 3CL | 3370 | 3378 | QPGQTFSVL | HLA-B*07:02 |  |  |  |
| YP_009724389.1 | 3CL | 3384 | 3393 | SPSGVYQCAM | HLA-B*07:02 |  |  |  |
| YP_009724389.1 | 3CL | 3392 | 3400 | AMRPNFTIK | HLA-A*03:01 |  |  |  |
| YP_009724389.1 | 3CL | 3394 | 3403 | RPNFTIKGSF | HLA-B*07:02 |  |  |  |
| YP_009724389.1 | 3CL | 3432 | 3445 | TGVHAGTDLEGNFY | HLA-A*01:01 |  |  |  |
| YP_009724389.1 | 3CL | 3482 | 3490 | FLNRFTTTL | HLA-A*02:01 |  |  |  |
| YP_009724389.1 | 3CL | 3523 | 3532 | AVLDMCASLK | HLA-A*11:01 |  |  |  |
| YP_009724389.1 | 3CL | 3548 | 3559 | ALLEDEFTPFDV | HLA-A*02:01 |  |  |  |
| YP_009724389.1 | 3CL | 3566 | 3574 | VTFQSAVKR | HLA-A*11:01 |  |  |  |
| YP_009724389.1 | nsp6 | 3587 | 3595 | ILTSLLVLV | HLA-A*02:01 |  |  |  |
| YP_009724389.1 | nsp6 | 3605 | 3616 | FLYENAFLPFAM | HLA-B*40:01 | HLA-A*02:01 | HLA-A*24:02 |  |
| YP_009724389.1 | nsp6 | 3612 | 3621 | LPFAMGIIAM | HLA-B*07:02 | HLA-B*35:01 |  |  |
| YP_009724389.1 | nsp6 | 3622 | 3630 | SAFAMMFVK | HLA-A*11:01 |  |  |  |
| YP_009724389.1 | nsp6 | 3628 | 3636 | FVKHKHAFL | HLA-B*08:01 |  |  |  |
| YP_009724389.1 | nsp6 | 3639 | 3649 | FLLPSLATVAY | HLA-B*35:01 | HLA-A*02:01 |  |  |
| YP_009724389.1 | nsp6 | 3653 | 3666 | VYMPASWVMRIMTW | HLA-A*23:01 | HLA-A*24:02 |  |  |
| YP_009724389.1 | nsp6 | 3666 | 3674 | WLDMVDTSL | HLA-A*02:01 |  |  |  |
| YP_009724389.1 | nsp6 | 3684 | 3692 | MYASAVVLL | HLA-A*23:01 | HLA-A*24:02 |  |  |
| YP_009724389.1 | nsp6 | 3703 | 3711 | DGARRVWTL | HLA-B*08:01 |  |  |  |
| YP_009724389.1 | nsp6 | 3710 | 3718 | TLMNVLTLV | HLA-A*02:01 |  |  |  |
| YP_009724389.1 | nsp6 | 3725 | 3733 | NALDQAISM | HLA-B*35:01 |  |  |  |
| YP_009724389.1 | nsp6 | 3732 | 3740 | SMWALIISV | HLA-A*02:01 |  |  |  |
| YP_009724389.1 | nsp6 | 3743 | 3753 | NYSGVVTTVMF | HLA-A*24:02 |  |  |  |
| YP_009724389.1 | nsp6 | 3752 | 3760 | MFLARGIVF | HLA-A*23:01 | HLA-A*24:02 | HLA-A*02:01 |  |
| YP_009724389.1 | nsp6 | 3768 | 3776 | IFFITGNTL | HLA-A*23:01 |  |  |  |
| YP_009724389.1 | nsp6 | 3811 | 3820 | YDYLVSTQEF | HLA-A*23:01 | HLA-A*24:02 |  |  |
| YP_009724389.1 | nsp7 | 3871 | 3879 | VLLSVLQQL | HLA-A*02:01 |  |  |  |
| YP_009724389.1 | nsp7 | 3886 | 3894 | KLWAQCVQL | HLA-A*02:01 |  |  |  |
| YP_009724389.1 | nsp7 | 3900 | 3908 | LAKDTTEAF | HLA-B*35:01 |  |  |  |
| YP_009724389.1 | nsp7 | 3905 | 3914 | TEAFEKMVSL | HLA-B*40:01 | HLA-B*08:01 |  |  |
| YP_009724389.1 | nsp7 | 3920 | 3929 | SMQGAVDINK | HLA-A*03:01 |  |  |  |
| YP_009724389.1 | nsp7 | 3933 | 3941 | EMLDNRATL | HLA-B*08:01 |  |  |  |
| YP_009724389.1 | nsp8 | 3945 | 3954 | ASEFSSLPSY | HLA-B*44:02 | HLA-B*44:03 |  |  |
| YP_009724389.1 | nsp8 | 3967 | 3978 | AVANGDSEVVLK | HLA-A*11:01 |  |  |  |
| YP_009724389.1 | nsp8 | 3976 | 3984 | VLKKLKKSL | HLA-B*08:01 |  |  |  |
| YP_009724389.1 | nsp8 | 3979 | 3988 | KLKKSLNVAK | HLA-A*03:01 |  |  |  |
| YP_009724389.1 | nsp8 | 3988 | 3997 | KSEFDRDAAM | HLA-B*40:01 | HLA-B*44:03 |  |  |
| YP_009724389.1 | nsp8 | 4004 | 4013 | MADQAMTQMY | HLA-A*01:01 | HLA-B*35:01 |  |  |
| YP_009724389.1 | nsp8 | 4030 | 4038 | QTMLFTMLR | HLA-A*11:01 |  |  |  |
| YP_009724389.1 | nsp8 | 4031 | 4039 | TMLFTMLRK | HLA-A*03:01 |  |  |  |
| YP_009724389.1 | nsp8 | 4062 | 4070 | IPLTTAAKL | HLA-B*07:02 |  |  |  |
| YP_009724389.1 | nsp8 | 4072 | 4081 | VVIPDYNTYK | HLA-A*03:01 | HLA-A*11:01 | HLA-B*35:01 |  |
| YP_009724389.1 | nsp8 | 4078 | 4091 | NTYKNTCDGTTFTY | HLA-A*01:01 |  |  |  |
| YP_009724389.1 | nsp8 | 4090 | 4098 | TYASALWEI | HLA-A*23:01 | HLA-A*24:02 |  |  |
| YP_009724389.1 | nsp8 | 4094 | 4102 | ALWEIQQVV | HLA-A*02:01 |  |  |  |
| YP_009724389.1 | nsp8 | 4112 | 4122 | SEISMDNSPNL | HLA-B*40:01 | HLA-B*44:02 | HLA-B*44:03 |  |
| YP_009724389.1 | nsp8 | 4116 | 4124 | MDNSPNLAW | HLA-B*44:02 | HLA-B*44:03 |  |  |
| YP_009724389.1 | nsp8 | 4119 | 4127 | SPNLAWPLI | HLA-B*07:02 |  |  |  |
| YP_009724389.1 | nsp8 | 4130 | 4138 | ALRANSAVK | HLA-A*03:01 |  |  |  |
| YP_009724389.1 | nsp9 | 4145 | 4152 | SPVALRQM | HLA-B*07:02 |  |  |  |
| YP_009724389.1 | nsp9 | 4158 | 4171 | TTQTACTDDNALAY | HLA-A*01:01 |  |  |  |
| YP_009724389.1 | nsp9 | 4160 | 4172 | QTACTDDNALAYY | HLA-A*01:01 |  |  |  |
| YP_009724389.1 | nsp9 | 4163 | 4172 | CTDDNALAYY | HLA-A*01:01 |  |  |  |
| YP_009724389.1 | nsp9 | 4168 | 4176 | ALAYYNTTK | HLA-A*03:01 | HLA-A*11:01 |  |  |
| YP_009724389.1 | nsp9 | 4183 | 4191 | ALLSDLQDL | HLA-A*02:01 |  |  |  |
| YP_009724389.1 | nsp9 | 4193 | 4206 | WARFPKSDGTGTIY | HLA-A*01:01 | HLA-B*07:02 | HLA-B*35:01 |  |
| YP_009724389.1 | nsp9 | 4207 | 4215 | TELEPPCRF | HLA-B*44:02 | HLA-B*44:03 |  |  |
| YP_009724389.1 | nsp9 | 4216 | 4229 | VTDTPKGPKVKYLY | HLA-A*01:01 |  |  |  |
| YP_009724389.1 | nsp9 | 4219 | 4228 | TPKGPKVKYL | HLA-B*07:02 | HLA-B*08:01 |  |  |
| YP_009724389.1 | nsp9 | 4222 | 4230 | GPKVKYLYF | HLA-B*08:01 |  |  |  |
| YP_009724389.1 | nsp9 | 4229 | 4237 | YFIKGLNNL | HLA-A*23:01 |  |  |  |
| YP_009724389.1 | nsp9 | 4235 | 4243 | NNLNRGMVL | HLA-B*08:01 |  |  |  |
| YP_009724389.1 | nsp10 | 4258 | 4267 | TEVPANSTVL | HLA-B*40:01 |  |  |  |
| YP_009724389.1 | nsp10 | 4260 | 4269 | VPANSTVLSF | HLA-B*07:02 |  |  |  |
| YP_009724389.1 | nsp10 | 4272 | 4281 | FAVDAAKAYK | HLA-B*35:01 | HLA-A*03:01 | HLA-A*11:01 |  |
| YP_009724389.1 | nsp10 | 4283 | 4291 | YLASGGQPI | HLA-A*02:01 |  |  |  |
| YP_009724389.1 | nsp10 | 4313 | 4321 | EANMDQESF | HLA-B*35:01 |  |  |  |
| YP_009724389.1 | nsp10 | 4336 | 4345 | HPNPKGFCDL | HLA-B*07:02 |  |  |  |
| YP_009724389.1 | nsp10 | 4344 | 4352 | DLKGKYVQI | HLA-B*08:01 |  |  |  |
| YP_009724389.1 | RdRpol | 4417 | 4425 | GTSTDVVYR | HLA-A*11:01 |  |  |  |
| YP_009724389.1 | RdRpol | 4419 | 4430 | STDVVYRAFDIY | HLA-A*01:01 |  |  |  |
| YP_009724389.1 | RdRpol | 4425 | 4433 | RAFDIYNDK | HLA-A*11:01 |  |  |  |
| YP_009724389.1 | RdRpol | 4429 | 4440 | IYNDKVAGFAKF | HLA-A*23:01 | HLA-A*24:02 |  |  |
| YP_009724389.1 | RdRpol | 4433 | 4442 | KVAGFAKFLK | HLA-A*03:01 | HLA-A*11:01 |  |  |
| YP_009724389.1 | RdRpol | 4456 | 4464 | NLIDSYFVV | HLA-A*02:01 |  |  |  |
| YP_009724389.1 | RdRpol | 4460 | 4469 | SYFVVKRHTF | HLA-A*23:01 | HLA-A*24:02 | HLA-B*08:01 |  |
| YP_009724389.1 | RdRpol | 4474 | 4482 | HEETIYNLL | HLA-B*40:01 |  |  |  |
| YP_009724389.1 | RdRpol | 4481 | 4490 | LLKDCPAVAK | HLA-A*03:01 |  |  |  |
| YP_009724389.1 | RdRpol | 4487 | 4495 | AVAKHDFFK | HLA-A*03:01 | HLA-A*11:01 |  |  |
| YP_009724389.1 | RdRpol | 4502 | 4511 | MVPHISRQRL | HLA-B*07:02 | HLA-B*08:01 |  |  |
| YP_009724389.1 | RdRpol | 4514 | 4523 | YTMADLVYAL | HLA-A*02:01 |  |  |  |
| YP_009724389.1 | RdRpol | 4557 | 4567 | FVENPDILRVY | HLA-A*01:01 | HLA-B*40:01 | HLA-B*44:02 |  |
| YP_009724389.1 | RdRpol | 4565 | 4573 | RVYANLGER | HLA-A*03:01 |  |  |  |
| YP_009724389.1 | RdRpol | 4631 | 4639 | SLLMPILTL | HLA-A*02:01 |  |  |  |
| YP_009724389.1 | RdRpol | 4634 | 4643 | MPILTLTRAL | HLA-B*07:02 |  |  |  |
| YP_009724389.1 | RdRpol | 4645 | 4653 | AESHVDTDL | HLA-B*40:01 |  |  |  |
| YP_009724389.1 | RdRpol | 4655 | 4663 | KPYIKWDLL | HLA-B*07:02 |  |  |  |
| YP_009724389.1 | RdRpol | 4673 | 4681 | KLFDRYFKY | HLA-A*03:01 | HLA-A*11:01 |  |  |
| YP_009724389.1 | RdRpol | 4711 | 4724 | TVFPPTSFGPLVRK | HLA-A*03:01 | HLA-A*11:01 |  |  |
| YP_009724389.1 | RdRpol | 4716 | 4724 | TSFGPLVRK | HLA-A*03:01 | HLA-A*11:01 |  |  |
| YP_009724389.1 | RdRpol | 4724 | 4733 | KIFVDGVPFV | HLA-A*02:01 |  |  |  |
| YP_009724389.1 | RdRpol | 4726 | 4738 | FVDGVPFVVSTGY | HLA-A*01:01 | HLA-A*02:01 |  |  |
| YP_009724389.1 | RdRpol | 4730 | 4738 | VPFVVSTGY | HLA-B*35:01 |  |  |  |
| YP_009724389.1 | RdRpol | 4801 | 4809 | TVKPGNFNK | HLA-A*03:01 | HLA-A*11:01 |  |  |
| YP_009724389.1 | RdRpol | 4806 | 4814 | NFNKDFYDF | HLA-A*23:01 | HLA-A*24:02 |  |  |
| YP_009724389.1 | RdRpol | 4839 | 4847 | NAAISDYDY | HLA-B*35:01 |  |  |  |
| YP_009724389.1 | RdRpol | 4841 | 4850 | AISDYDYYRY | HLA-A*01:01 |  |  |  |
| YP_009724389.1 | RdRpol | 4863 | 4871 | FVVEVVDKY | HLA-B*35:01 |  |  |  |
| YP_009724389.1 | RdRpol | 4867 | 4875 | VVDKYFDCY | HLA-A*01:01 |  |  |  |
| YP_009724389.1 | RdRpol | 4892 | 4900 | KSAGFPFNK | HLA-A*03:01 | HLA-A*11:01 |  |  |
| YP_009724389.1 | RdRpol | 4905 | 4913 | RLYYDSMSY | HLA-A*03:01 | HLA-A*11:01 |  |  |
| YP_009724389.1 | RdRpol | 4912 | 4922 | SYEDQDALFAY | HLA-B*44:03 | HLA-A*23:01 | HLA-A*24:02 |  |
| YP_009724389.1 | RdRpol | 4920 | 4928 | FAYTKRNVI | HLA-B*08:01 |  |  |  |
| YP_009724389.1 | RdRpol | 4928 | 4936 | IPTITQMNL | HLA-B*07:02 |  |  |  |
| YP_009724389.1 | RdRpol | 4941 | 4949 | SAKNRARTV | HLA-B*08:01 |  |  |  |
| YP_009724389.1 | RdRpol | 4958 | 4969 | MTNRQFHQKLLK | HLA-A*03:01 | HLA-A*11:01 |  |  |
| YP_009724389.1 | RdRpol | 4971 | 4979 | IAATRGATV | HLA-B*08:01 |  |  |  |
| YP_009724389.1 | RdRpol | 4977 | 4985 | ATVVIGTSK | HLA-A*03:01 | HLA-A*11:01 |  |  |
| YP_009724389.1 | RdRpol | 4986 | 4994 | FYGGWHNML | HLA-A*24:02 | HLA-A*23:01 |  |  |
| YP_009724389.1 | RdRpol | 4996 | 5009 | TVYSDVENPHLMGW | HLA-B*44:02 |  |  |  |
| YP_009724389.1 | RdRpol | 4998 | 5011 | YSDVENPHLMGWDY | HLA-A*01:01 |  |  |  |
| YP_009724389.1 | RdRpol | 4999 | 5009 | SDVENPHLMGW | HLA-B*44:02 | HLA-B*44:03 |  |  |
| YP_009724389.1 | RdRpol | 5003 | 5011 | NPHLMGWDY | HLA-B*35:01 |  |  |  |
| YP_009724389.1 | RdRpol | 5039 | 5047 | SLSHRFYRL | HLA-B*08:01 |  |  |  |
| YP_009724389.1 | RdRpol | 5046 | 5054 | RLANECAQV | HLA-A*02:01 |  |  |  |
| YP_009724389.1 | RdRpol | 5055 | 5066 | LSEMVMCGGSLY | HLA-A*01:01 | HLA-B*44:02 | HLA-B*44:03 |  |
| YP_009724389.1 | RdRpol | 5080 | 5088 | AYANSVFNI | HLA-A*23:01 | HLA-A*24:02 |  |  |
| YP_009724389.1 | RdRpol | 5101 | 5111 | STDGNKIADKY | HLA-A*01:01 |  |  |  |
| YP_009724389.1 | RdRpol | 5126 | 5138 | NRDVDTDFVNEFY | HLA-A*01:01 |  |  |  |
| YP_009724389.1 | RdRpol | 5130 | 5140 | DTDFVNEFYAY | HLA-A*01:01 | HLA-A*02:01 |  |  |
| YP_009724389.1 | RdRpol | 5138 | 5145 | YAYLRKHF | HLA-B*08:01 |  |  |  |
| YP_009724389.1 | RdRpol | 5140 | 5148 | YLRKHFSMM | HLA-B*08:01 |  |  |  |
| YP_009724389.1 | RdRpol | 5150 | 5162 | LSDDAVVCFNSTY | HLA-A*01:01 |  |  |  |
| YP_009724389.1 | RdRpol | 5167 | 5175 | LVASIKNFK | HLA-A*03:01 | HLA-A*11:01 |  |  |
| YP_009724389.1 | RdRpol | 5170 | 5178 | SIKNFKSVL | HLA-B*08:01 |  |  |  |
| YP_009724389.1 | RdRpol | 5202 | 5211 | HEFCSQHTML | HLA-B*40:01 |  |  |  |
| YP_009724389.1 | RdRpol | 5220 | 5230 | YLPYPDPSRIL | HLA-A*02:01 | HLA-B*07:02 |  |  |
| YP_009724389.1 | RdRpol | 5246 | 5254 | LMIERFVSL | HLA-A*02:01 | HLA-B*08:01 |  |  |
| YP_009724389.1 | RdRpol | 5251 | 5259 | FVSLAIDAY | HLA-B*35:01 |  |  |  |
| YP_009724389.1 | RdRpol | 5255 | 5263 | AIDAYPLTK | HLA-A*03:01 | HLA-A*11:01 |  |  |
| YP_009724389.1 | RdRpol | 5259 | 5269 | YPLTKHPNQEY | HLA-B*35:01 |  |  |  |
| YP_009724389.1 | RdRpol | 5264 | 5276 | HPNQEYADVFHLY | HLA-B*35:01 | HLA-B*40:01 | HLA-B*44:02 | HLA-B*44:03 |
| YP_009724389.1 | RdRpol | 5269 | 5276 | YADVFHLY | HLA-A*01:01 |  |  |  |
| YP_009724389.1 | RdRpol | 5274 | 5283 | HLYLQYIRKL | HLA-A*23:01 | HLA-A*24:02 | HLA-A*03:01 |  |
| YP_009724389.1 | RdRpol | 5291 | 5299 | MLDMYSVML | HLA-A*02:01 |  |  |  |
| YP_009724389.1 | RdRpol | 5295 | 5307 | YSVMLTNDNTSRY | HLA-A*01:01 |  |  |  |
| YP_009724389.1 | RdRpol | 5313 | 5323 | YEAMYTPHTVL | HLA-B*40:01 |  |  |  |
| YP_009724389.1 | Hel | 5318 | 5326 | TPHTVLQAV | HLA-B*07:02 |  |  |  |
| YP_009724389.1 | Hel | 5380 | 5394 | DVTDVTQLYLGGMSY | HLA-A*01:01 | HLA-A*03:01 | HLA-B*35:01 |  |
| YP_009724389.1 | Hel | 5393 | 5405 | SYYCKSHKPPISF | HLA-A*23:01 | HLA-A*24:02 |  |  |
| YP_009724389.1 | Hel | 5405 | 5414 | FPLCANGQVF | HLA-B*35:01 |  |  |  |
| YP_009724389.1 | Hel | 5455 | 5463 | KLFAAETLK | HLA-A*03:01 | HLA-A*11:01 |  |  |
| YP_009724389.1 | Hel | 5464 | 5473 | ATEETFKLSY | HLA-A*01:01 | HLA-B*44:02 | HLA-B*44:03 |  |
| YP_009724389.1 | Hel | 5470 | 5478 | KLSYGIATV | HLA-A*02:01 |  |  |  |
| YP_009724389.1 | Hel | 5479 | 5491 | REVLSDRELHLSW | HLA-B*44:02 | HLA-B*40:01 | HLA-A*02:01 |  |
| YP_009724389.1 | Hel | 5483 | 5493 | SDRELHLSWEV | HLA-B*40:01 | HLA-B*44:02 |  |  |
| YP_009724389.1 | Hel | 5493 | 5500 | VGKPRPPL | HLA-B*08:01 |  |  |  |
| YP_009724389.1 | Hel | 5495 | 5506 | KPRPPLNRNYVF | HLA-B*07:02 |  |  |  |
| YP_009724389.1 | Hel | 5504 | 5513 | YVFTGYRVTK | HLA-A*03:01 | HLA-A*11:01 |  |  |
| YP_009724389.1 | Hel | 5520 | 5529 | GEYTFEKGDY | HLA-B*44:02 | HLA-B*44:03 |  |  |
| YP_009724389.1 | Hel | 5533 | 5542 | VVYRGTTTYK | HLA-A*03:01 | HLA-A*11:01 | HLA-B*35:01 |  |
| YP_009724389.1 | Hel | 5540 | 5550 | TYKLNVGDYFV | HLA-A*23:01 | HLA-A*24:02 | HLA-A*02:01 |  |
| YP_009724389.1 | Hel | 5557 | 5564 | MPLSAPTL | HLA-B*07:02 |  |  |  |
| YP_009724389.1 | Hel | 5563 | 5571 | TLVPQEHYV | HLA-A*02:01 |  |  |  |
| YP_009724389.1 | Hel | 5577 | 5586 | YPTLNISDEF | HLA-B*35:01 |  |  |  |
| YP_009724389.1 | Hel | 5582 | 5593 | ISDEFSSNVANY | HLA-A*01:01 | HLA-B*44:03 |  |  |
| YP_009724389.1 | Hel | 5587 | 5595 | SSNVANYQK | HLA-A*11:01 |  |  |  |
| YP_009724389.1 | Hel | 5596 | 5604 | VGMQKYSTL | HLA-B*08:01 |  |  |  |
| YP_009724389.1 | Hel | 5602 | 5612 | STLQGPPGTGK | HLA-A*11:01 | HLA-A*03:01 |  |  |
| YP_009724389.1 | Hel | 5615 | 5623 | FAIGLALYY | HLA-B*35:01 |  |  |  |
| YP_009724389.1 | Hel | 5623 | 5630 | YPSARIVY | HLA-B*35:01 |  |  |  |
| YP_009724389.1 | Hel | 5645 | 5653 | ALKYLPIDK | HLA-A*03:01 |  |  |  |
| YP_009724389.1 | Hel | 5658 | 5667 | IPARARVECF | HLA-B*07:02 |  |  |  |
| YP_009724389.1 | Hel | 5698 | 5706 | DEISMATNY | HLA-B*44:02 | HLA-B*44:03 |  |  |
| YP_009724389.1 | Hel | 5710 | 5718 | VVNARLRAK | HLA-A*03:01 |  |  |  |
| YP_009724389.1 | Hel | 5721 | 5729 | VYIGDPAQL | HLA-A*23:01 | HLA-A*24:02 |  |  |
| YP_009724389.1 | Hel | 5731 | 5741 | APRTLLTKGTL | HLA-B*07:02 | HLA-B*08:01 |  |  |
| YP_009724389.1 | Hel | 5770 | 5781 | AEIVDTVSALVY | HLA-B*44:03 | HLA-B*40:01 | HLA-B*44:02 | HLA-A*01:01 |
| YP_009724389.1 | Hel | 5814 | 5823 | RPQIGVVREF | HLA-B*07:02 |  |  |  |
| YP_009724389.1 | Hel | 5821 | 5830 | REFLTRNPAW | HLA-B*44:02 | HLA-B*44:03 |  |  |
| YP_009724389.1 | Hel | 5826 | 5835 | RNPAWRKAVF | HLA-B*07:02 | HLA-B*08:01 |  |  |
| YP_009724389.1 | Hel | 5837 | 5850 | SPYNSQNAVASKIL | HLA-B*07:02 |  |  |  |
| YP_009724389.1 | Hel | 5856 | 5865 | TVDSSQGSEY | HLA-A*01:01 |  |  |  |
| YP_009724389.1 | Hel | 5906 | 5914 | YDKLQFTSL | HLA-B*08:01 |  |  |  |
| YP_009724389.1 | Hel | 5916 | 5924 | IPRRNVATL | HLA-B*07:02 | HLA-B*08:01 |  |  |
| YP_009724389.1 | nsp14 | 5926 | 5933 | AENVTGLF | HLA-B*44:02 |  |  |  |
| YP_009724389.1 | nsp14 | 5944 | 5952 | HPTQAPTHL | HLA-B*07:02 | HLA-B*35:01 |  |  |
| YP_009724389.1 | nsp14 | 5967 | 5976 | IPGIPKDMTY | HLA-B*35:01 |  |  |  |
| YP_009724389.1 | nsp14 | 5974 | 5982 | MTYRRLISM | HLA-B*08:01 |  |  |  |
| YP_009724389.1 | nsp14 | 5978 | 5986 | RLISMMGFK | HLA-A*03:01 |  |  |  |
| YP_009724389.1 | nsp14 | 5981 | 5989 | SMMGFKMNY | HLA-A*03:01 | HLA-A*11:01 |  |  |
| YP_009724389.1 | nsp14 | 6001 | 6011 | REEAIRHVRAW | HLA-B*44:02 | HLA-B*44:03 |  |  |
| YP_009724389.1 | nsp14 | 6023 | 6032 | REAVGTNLPL | HLA-B*40:01 |  |  |  |
| YP_009724389.1 | nsp14 | 6086 | 6094 | VVRIKIVQM | HLA-B*08:01 |  |  |  |
| YP_009724389.1 | nsp14 | 6094 | 6102 | MLSDTLKNL | HLA-A*02:01 |  |  |  |
| YP_009724389.1 | nsp14 | 6101 | 6109 | NLSDRVVFV | HLA-A*02:01 |  |  |  |
| YP_009724389.1 | nsp14 | 6107 | 6115 | VFVLWAHGF | HLA-A*23:01 |  |  |  |
| YP_009724389.1 | nsp14 | 6109 | 6117 | VLWAHGFEL | HLA-A*02:01 |  |  |  |
| YP_009724389.1 | nsp14 | 6115 | 6123 | FELTSMKYF | HLA-B*44:02 | HLA-B*44:03 |  |  |
| YP_009724389.1 | nsp14 | 6154 | 6162 | HSIGFDYVY | HLA-B*35:01 |  |  |  |
| YP_009724389.1 | nsp14 | 6159 | 6167 | DYVYNPFMI | HLA-A*24:02 |  |  |  |
| YP_009724389.1 | nsp14 | 6195 | 6203 | ASCDAIMTR | HLA-A*11:01 |  |  |  |
| YP_009724389.1 | nsp14 | 6219 | 6228 | IEYPIIGDEL | HLA-B*40:01 |  |  |  |
| YP_009724389.1 | nsp14 | 6239 | 6246 | HMVVKAAL | HLA-B*08:01 |  |  |  |
| YP_009724389.1 | nsp14 | 6246 | 6254 | LLADKFPVL | HLA-A*02:01 | HLA-B*08:01 |  |  |
| YP_009724389.1 | nsp14 | 6253 | 6261 | VLHDIGNPK | HLA-A*03:01 |  |  |  |
| YP_009724389.1 | nsp14 | 6294 | 6302 | SYATHSDKF | HLA-A*23:01 | HLA-A*24:02 |  |  |
| YP_009724389.1 | nsp14 | 6301 | 6309 | KFTDGVCLF | HLA-A*23:01 | HLA-A*24:02 |  |  |
| YP_009724389.1 | nsp14 | 6317 | 6326 | YPANSIVCRF | HLA-B*35:01 |  |  |  |
| YP_009724389.1 | nsp14 | 6343 | 6351 | SLYVNKHAF | HLA-B*08:01 |  |  |  |
| YP_009724389.1 | nsp14 | 6353 | 6361 | TPAFDKSAF | HLA-B*07:02 | HLA-B*35:01 |  |  |
| YP_009724389.1 | nsp14 | 6412 | 6420 | HANEYRLYL | HLA-B*08:01 |  |  |  |
| YP_009724389.1 | nsp14 | 6418 | 6426 | LYLDAYNMM | HLA-A*23:01 | HLA-A*24:02 |  |  |
| YP_009724389.1 | nsp14 | 6419 | 6427 | YLDAYNMMI | HLA-A*02:01 |  |  |  |
| YP_009724389.1 | nsp14 | 6425 | 6433 | MMISAGFSL | HLA-A*02:01 |  |  |  |
| YP_009724389.1 | nsp14 | 6435 | 6445 | VYKQFDTYNLW | HLA-A*23:01 | HLA-A*24:02 |  |  |
| YP_009724389.1 | nsp14 | 6441 | 6448 | TYNLWNTF | HLA-A*24:02 |  |  |  |
| YP_009724389.1 | nsp14 | 6446 | 6454 | NTFTRLQSL | HLA-B*08:01 |  |  |  |
| YP_009724389.1 | nsp15 | 6453 | 6461 | SLENVAFNV | HLA-A*02:01 |  |  |  |
| YP_009724389.1 | nsp15 | 6477 | 6486 | SIINNTVYTK | HLA-A*03:01 | HLA-A*11:01 |  |  |
| YP_009724389.1 | nsp15 | 6492 | 6501 | VELFENKTTL | HLA-B*40:01 | HLA-B*08:01 |  |  |
| YP_009724389.1 | nsp15 | 6501 | 6509 | LPVNVAFEL | HLA-B*07:02 | HLA-B*35:01 |  |  |
| YP_009724389.1 | nsp15 | 6516 | 6524 | KPVPEVKIL | HLA-B*07:02 |  |  |  |
| YP_009724389.1 | nsp15 | 6544 | 6551 | APAHISTI | HLA-B*07:02 |  |  |  |
| YP_009724389.1 | nsp15 | 6573 | 6586 | VFFDGRVDGQVDLF | HLA-A*23:01 |  |  |  |
| YP_009724389.1 | nsp15 | 6602 | 6610 | GLQPSVGPK | HLA-A*03:01 |  |  |  |
| YP_009724389.1 | nsp15 | 6606 | 6614 | SVGPKQASL | HLA-B*08:01 |  |  |  |
| YP_009724389.1 | nsp15 | 6608 | 6619 | GPKQASLNGVTL | HLA-B*07:02 |  |  |  |
| YP_009724389.1 | nsp15 | 6621 | 6630 | GEAVKTQFNY | HLA-B*44:02 | HLA-B*44:03 |  |  |
| YP_009724389.1 | nsp15 | 6622 | 6630 | EAVKTQFNY | HLA-B*35:01 |  |  |  |
| YP_009724389.1 | nsp15 | 6625 | 6633 | KTQFNYYKK | HLA-A*11:01 |  |  |  |
| YP_009724389.1 | nsp15 | 6653 | 6661 | QEFKPRSQM | HLA-B*40:01 | HLA-B*44:02 | HLA-B*44:03 | HLA-B*08:01 |
| YP_009724389.1 | nsp15 | 6656 | 6663 | KPRSQMEI | HLA-B*07:02 |  |  |  |
| YP_009724389.1 | nsp15 | 6661 | 6670 | MEIDFLELAM | HLA-B*40:01 |  |  |  |
| YP_009724389.1 | nsp15 | 6668 | 6677 | LAMDEFIERY | HLA-A*01:01 |  |  |  |
| YP_009724389.1 | nsp15 | 6676 | 6684 | RYKLEGYAF | HLA-A*23:01 | HLA-A*24:02 |  |  |
| YP_009724389.1 | nsp15 | 6682 | 6689 | YAFEHIVY | HLA-B*35:01 |  |  |  |
| YP_009724389.1 | nsp15 | 6695 | 6703 | SQLGGLHLL | HLA-A*02:01 |  |  |  |
| YP_009724389.1 | nsp15 | 6715 | 6723 | FELEDFIPM | HLA-B*40:01 |  |  |  |
| YP_009724389.1 | nsp15 | 6721 | 6730 | IPMDSTVKNY | HLA-B*35:01 |  |  |  |
| YP_009724389.1 | nsp15 | 6749 | 6758 | LLLDDFVEII | HLA-A*02:01 |  |  |  |
| YP_009724389.1 | nsp15 | 6755 | 6763 | VEIIKSQDL | HLA-B*40:01 |  |  |  |
| YP_009724389.1 | nsp15 | 6777 | 6784 | TEISFMLW | HLA-B*44:02 | HLA-B*44:03 |  |  |
| YP_009724389.1 | nsp16 | 6801 | 6809 | QAWQPGVAM | HLA-B*35:01 |  |  |  |
| YP_009724389.1 | nsp16 | 6806 | 6815 | GVAMPNLYKM | HLA-A*03:01 | HLA-A*11:01 | HLA-B*35:01 |  |
| YP_009724389.1 | nsp16 | 6814 | 6822 | KMQRMLLEK | HLA-A*03:01 |  |  |  |
| YP_009724389.1 | nsp16 | 6834 | 6842 | LPKGIMMNV | HLA-B*07:02 |  |  |  |
| YP_009724389.1 | nsp16 | 6841 | 6848 | NVAKYTQL | HLA-B*08:01 |  |  |  |
| YP_009724389.1 | nsp16 | 6851 | 6859 | YLNTLTLAV | HLA-A*02:01 |  |  |  |
| YP_009724389.1 | nsp16 | 6859 | 6868 | VPYNMRVIHF | HLA-B*07:02 | HLA-A*23:01 | HLA-A*24:02 |  |
| YP_009724389.1 | nsp16 | 6908 | 6916 | TLIGDCATV | HLA-A*02:01 |  |  |  |
| YP_009724389.1 | nsp16 | 6939 | 6950 | KENDSKEGFFTY | HLA-B*44:02 | HLA-B*44:03 | HLA-B*35:01 |  |
| YP_009724389.1 | nsp16 | 6954 | 6961 | FIQQKLAL | HLA-B*08:01 |  |  |  |
| YP_009724389.1 | nsp16 | 6960 | 6968 | ALGGSVAIK | HLA-A*03:01 |  |  |  |
| YP_009724389.1 | nsp16 | 6969 | 6979 | ITEHSWNADLY | HLA-A*01:01 | HLA-B*40:01 |  |  |
| YP_009724389.1 | nsp16 | 6972 | 6980 | HSWNADLYK | HLA-A*11:01 |  |  |  |
| YP_009724389.1 | nsp16 | 7012 | 7022 | KPREQIDGYVM | HLA-B*07:02 | HLA-B*35:01 | HLA-B*40:01 |  |
| YP_009724389.1 | nsp16 | 7038 | 7048 | SSYSLFDMSK | HLA-A*11:01 | HLA-A*23:01 | HLA-A*24:02 |  |
| YP_009724389.1 | nsp16 | 7041 | 7052 | SLFDMSKFPLKL | HLA-A*02:01 |  |  |  |
| YP_009724390.1 | surface glycoprotein | 17 | 24 | NLTTRTQL | HLA-B*08:01 |  |  |  |
| YP_009724390.1 | surface glycoprotein | 24 | 32 | LPPAYTNSF | HLA-B*35:01 |  |  |  |
| YP_009724390.1 | surface glycoprotein | 41 | 49 | KVFRSSVLH | HLA-A*03:01 |  |  |  |
| YP_009724390.1 | surface glycoprotein | 56 | 64 | LPFFSNVTW | HLA-B*35:01 |  |  |  |
| YP_009724390.1 | surface glycoprotein | 57 | 65 | PFFSNVTWF | HLA-A*23:01 | HLA-A*24:02 |  |  |
| YP_009724390.1 | surface glycoprotein | 78 | 86 | RFDNPVLPF | HLA-A*23:01 | HLA-A*24:02 |  |  |
| YP_009724390.1 | surface glycoprotein | 84 | 92 | LPFNDGVYF | HLA-B*07:02 | HLA-B*35:01 |  |  |
| YP_009724390.1 | surface glycoprotein | 89 | 97 | GVYFASTEK | HLA-A*03:01 | HLA-A*11:01 |  |  |
| YP_009724390.1 | surface glycoprotein | 95 | 104 | TEKSNIIRGW | HLA-B*44:02 | HLA-B*44:03 |  |  |
| YP_009724390.1 | surface glycoprotein | 109 | 117 | TLDSKTQSL | HLA-B*08:01 |  |  |  |
| YP_009724390.1 | surface glycoprotein | 142 | 150 | GVYYHKNNK | HLA-A*03:01 |  |  |  |
| YP_009724390.1 | surface glycoprotein | 144 | 152 | YYHKNNKSW | HLA-A*24:02 |  |  |  |
| YP_009724390.1 | surface glycoprotein | 159 | 168 | VYSSANNCTF | HLA-A*23:01 | HLA-A*24:02 |  |  |
| YP_009724390.1 | surface glycoprotein | 168 | 176 | FEYVSQPFL | HLA-B*40:01 |  |  |  |
| YP_009724390.1 | surface glycoprotein | 169 | 177 | EYVSQPFLM | HLA-A*23:01 | HLA-A*24:02 |  |  |
| YP_009724390.1 | surface glycoprotein | 192 | 200 | FVFKNIDGY | HLA-B*35:01 |  |  |  |
| YP_009724390.1 | surface glycoprotein | 208 | 216 | TPINLVRDL | HLA-B*07:02 |  |  |  |
| YP_009724390.1 | surface glycoprotein | 216 | 223 | LPQGFSAL | HLA-B*07:02 |  |  |  |
| YP_009724390.1 | surface glycoprotein | 229 | 238 | LPIGINITRF | HLA-B*35:01 |  |  |  |
| YP_009724390.1 | surface glycoprotein | 233 | 241 | INITRFQTL | HLA-B*08:01 |  |  |  |
| YP_009724390.1 | surface glycoprotein | 241 | 249 | LLALHRSYL | HLA-B*08:01 |  |  |  |
| YP_009724390.1 | surface glycoprotein | 258 | 266 | WTAGAAAYY | HLA-A*01:01 |  |  |  |
| YP_009724390.1 | surface glycoprotein | 265 | 275 | YYVGYLQPRTF | HLA-A*23:01 | HLA-A*24:02 |  |  |
| YP_009724390.1 | surface glycoprotein | 269 | 277 | YLQPRTFLL | HLA-A*02:01 | HLA-B*08:01 |  |  |
| YP_009724390.1 | surface glycoprotein | 269 | 276 | YLQPRTFL | HLA-B*08:01 |  |  |  |
| YP_009724390.1 | surface glycoprotein | 297 | 306 | SETKCTLKSF | HLA-B*44:02 | HLA-B*44:03 |  |  |
| YP_009724390.1 | surface glycoprotein | 302 | 310 | TLKSFTVEK | HLA-A*03:01 | HLA-A*11:01 |  |  |
| YP_009724390.1 | surface glycoprotein | 321 | 329 | QPTESIVRF | HLA-B*35:01 |  |  |  |
| YP_009724390.1 | surface glycoprotein | 328 | 338 | RFPNITNLCPF | HLA-A*24:02 |  |  |  |
| YP_009724390.1 | surface glycoprotein | 339 | 347 | GEVFNATRF | HLA-B*40:01 | HLA-B*44:02 | HLA-B*44:03 |  |
| YP_009724390.1 | surface glycoprotein | 343 | 351 | NATRFASVY | HLA-B*35:01 |  |  |  |
| YP_009724390.1 | surface glycoprotein | 368 | 377 | LYNSASFSTF | HLA-A*23:01 | HLA-A*24:02 |  |  |
| YP_009724390.1 | surface glycoprotein | 370 | 378 | NSASFSTFK | HLA-A*11:01 |  |  |  |
| YP_009724390.1 | surface glycoprotein | 408 | 417 | RQIAPGQTGK | HLA-A*03:01 |  |  |  |
| YP_009724390.1 | surface glycoprotein | 417 | 425 | KIADYNYKL | HLA-A*02:01 |  |  |  |
| YP_009724390.1 | surface glycoprotein | 448 | 456 | NYNYLYRLF | HLA-A*23:01 | HLA-A*24:02 |  |  |
| YP_009724390.1 | surface glycoprotein | 454 | 462 | RLFRKSNLK | HLA-A*03:01 |  |  |  |
| YP_009724390.1 | surface glycoprotein | 462 | 472 | KPFERDISTEI | HLA-B*07:02 | HLA-B*40:01 |  |  |
| YP_009724390.1 | surface glycoprotein | 489 | 497 | YFPLQSYGF | HLA-A*23:01 | HLA-A*24:02 |  |  |
| YP_009724390.1 | surface glycoprotein | 506 | 513 | QPYRVVVL | HLA-B*07:02 | HLA-B*08:01 |  |  |
| YP_009724390.1 | surface glycoprotein | 507 | 515 | PYRVVVLSF | HLA-A*23:01 | HLA-A*24:02 |  |  |
| YP_009724390.1 | surface glycoprotein | 526 | 534 | GPKKSTNLV | HLA-B*07:02 | HLA-B*08:01 |  |  |
| YP_009724390.1 | surface glycoprotein | 604 | 612 | TSNQVAVLY | HLA-A*01:01 |  |  |  |
| YP_009724390.1 | surface glycoprotein | 635 | 643 | VYSTGSNVF | HLA-A*23:01 | HLA-A*24:02 |  |  |
| YP_009724390.1 | surface glycoprotein | 653 | 660 | AEHVNNSY | HLA-B*44:03 |  |  |  |
| YP_009724390.1 | surface glycoprotein | 664 | 674 | IPIGAGICASY | HLA-B*35:01 |  |  |  |
| YP_009724390.1 | surface glycoprotein | 680 | 688 | SPRRARSVA | HLA-B*07:02 |  |  |  |
| YP_009724390.1 | surface glycoprotein | 687 | 695 | VASQSIIAY | HLA-B*35:01 |  |  |  |
| YP_009724390.1 | surface glycoprotein | 691 | 699 | SIIAYTMSL | HLA-A*02:01 |  |  |  |
| YP_009724390.1 | surface glycoprotein | 699 | 707 | LGAENSVAY | HLA-B*35:01 |  |  |  |
| YP_009724390.1 | surface glycoprotein | 706 | 718 | AYSNNSIAIPTNF | HLA-A*24:02 |  |  |  |
| YP_009724390.1 | surface glycoprotein | 714 | 722 | IPTNFTISV | HLA-B*07:02 |  |  |  |
| YP_009724390.1 | surface glycoprotein | 724 | 733 | TEILPVSMTK | HLA-A*11:01 |  |  |  |
| YP_009724390.1 | surface glycoprotein | 779 | 789 | QEVFAQVKQIY | HLA-B*44:02 | HLA-B*44:03 |  |  |
| YP_009724390.1 | surface glycoprotein | 786 | 795 | KQIYKTPPIK | HLA-A*03:01 |  |  |  |
| YP_009724390.1 | surface glycoprotein | 788 | 797 | IYKTPPIKDF | HLA-A*23:01 | HLA-A*24:02 |  |  |
| YP_009724390.1 | surface glycoprotein | 821 | 829 | LLFNKVTLA | HLA-A*02:01 | HLA-B*08:01 |  |  |
| YP_009724390.1 | surface glycoprotein | 827 | 835 | TLADAGFIK | HLA-A*03:01 | HLA-A*11:01 |  |  |
| YP_009724390.1 | surface glycoprotein | 828 | 837 | LADAGFIKQY | HLA-A*01:01 |  |  |  |
| YP_009724390.1 | surface glycoprotein | 829 | 837 | ADAGFIKQY | HLA-B*44:02 | HLA-B*44:03 |  |  |
| YP_009724390.1 | surface glycoprotein | 860 | 873 | VLPPLLTDEMIAQY | HLA-A*01:01 |  |  |  |
| YP_009724390.1 | surface glycoprotein | 896 | 904 | IPFAMQMAY | HLA-B*35:01 |  |  |  |
| YP_009724390.1 | surface glycoprotein | 939 | 947 | SSTASALGK | HLA-A*11:01 |  |  |  |
| YP_009724390.1 | surface glycoprotein | 976 | 984 | VLNDILSRL | HLA-A*02:01 | HLA-A*11:01 |  |  |
| YP_009724390.1 | surface glycoprotein | 983 | 991 | RLDKVEAEV | HLA-A*02:01 |  |  |  |
| YP_009724390.1 | surface glycoprotein | 987 | 996 | VEAEVQIDRL | HLA-B*40:01 |  |  |  |
| YP_009724390.1 | surface glycoprotein | 989 | 997 | AEVQIDRLI | HLA-B*40:01 | HLA-B*44:02 |  |  |
| YP_009724390.1 | surface glycoprotein | 989 | 997 | AEVQIDRLI | HLA-B*44:03 |  |  |  |
| YP_009724390.1 | surface glycoprotein | 996 | 1004 | LITGRLQSL | HLA-B*08:01 |  |  |  |
| YP_009724390.1 | surface glycoprotein | 1000 | 1008 | RLQSLQTYV | HLA-A*02:01 |  |  |  |
| YP_009724390.1 | surface glycoprotein | 1016 | 1024 | AEIRASANL | HLA-B*40:01 | HLA-B*44:02 | HLA-B*44:03 |  |
| YP_009724390.1 | surface glycoprotein | 1020 | 1028 | ASANLAATK | HLA-A*03:01 | HLA-A*11:01 |  |  |
| YP_009724390.1 | surface glycoprotein | 1048 | 1056 | HLMSFPQSA | HLA-A*02:01 |  |  |  |
| YP_009724390.1 | surface glycoprotein | 1052 | 1062 | FPQSAPHGVVF | HLA-B*35:01 |  |  |  |
| YP_009724390.1 | surface glycoprotein | 1056 | 1063 | APHGVVFL | HLA-B*07:02 |  |  |  |
| YP_009724390.1 | surface glycoprotein | 1065 | 1073 | VTYVPAQEK | HLA-A*03:01 | HLA-A*11:01 |  |  |
| YP_009724390.1 | surface glycoprotein | 1066 | 1075 | TYVPAQEKNF | HLA-A*23:01 | HLA-A*24:02 |  |  |
| YP_009724390.1 | surface glycoprotein | 1091 | 1102 | REGVFVSNGTHW | HLA-B*44:02 | HLA-B*44:03 | HLA-A*23:01 | HLA-A*24:02 |
| YP_009724390.1 | surface glycoprotein | 1099 | 1107 | GTHWFVTQR | HLA-A*11:01 |  |  |  |
| YP_009724390.1 | surface glycoprotein | 1136 | 1149 | TVYDPLQPELDSFK | HLA-A*11:01 | HLA-A*23:01 |  |  |
| YP_009724390.1 | surface glycoprotein | 1181 | 1189 | KEIDRLNEV | HLA-B*40:01 |  |  |  |
| YP_009724390.1 | surface glycoprotein | 1201 | 1212 | QELGKYEQYIKW | HLA-B*44:02 | HLA-B*44:03 |  |  |
| YP_009724390.1 | surface glycoprotein | 1206 | 1214 | YEQYIKWPW | HLA-B*44:02 | HLA-B*44:03 |  |  |
| YP_009724390.1 | surface glycoprotein | 1208 | 1216 | QYIKWPWYI | HLA-A*23:01 | HLA-A*24:02 |  |  |
| YP_009724390.1 | surface glycoprotein | 1220 | 1228 | FIAGLIAIV | HLA-A*02:01 |  |  |  |
| YP_009724390.1 | surface glycoprotein | 1261 | 1270 | SEPVLKGVKL | HLA-B*07:02 | HLA-B*08:01 |  |  |
| YP_009724391.1 | ORF3a protein | 6 | 16 | RIFTIGTVTLK | HLA-A*03:01 | HLA-A*11:01 |  |  |
| YP_009724391.1 | ORF3a protein | 35 | 43 | IPIQASLPF | HLA-B*07:02 | HLA-B*35:01 |  |  |
| YP_009724391.1 | ORF3a protein | 58 | 66 | SASKIITLK | HLA-A*03:01 | HLA-A*11:01 | HLA-B*08:01 |  |
| YP_009724391.1 | ORF3a protein | 59 | 67 | ASKIITLKK | HLA-A*03:01 | HLA-A*11:01 |  |  |
| YP_009724391.1 | ORF3a protein | 63 | 71 | ITLKKRWQL | HLA-B*08:01 |  |  |  |
| YP_009724391.1 | ORF3a protein | 72 | 80 | ALSKGVHFV | HLA-A*02:01 |  |  |  |
| YP_009724391.1 | ORF3a protein | 89 | 97 | TVYSHLLLV | HLA-A*02:01 |  |  |  |
| YP_009724391.1 | ORF3a protein | 100 | 109 | GLEAPFLYLY | HLA-B*44:02 | HLA-B*44:03 |  |  |
| YP_009724391.1 | ORF3a protein | 103 | 111 | APFLYLYAL | HLA-B*07:02 |  |  |  |
| YP_009724391.1 | ORF3a protein | 106 | 114 | LYLYALVYF | HLA-A*23:01 | HLA-A*24:02 |  |  |
| YP_009724391.1 | ORF3a protein | 107 | 115 | YLYALVYFL | HLA-A*02:01 |  |  |  |
| YP_009724391.1 | ORF3a protein | 112 | 120 | VYFLQSINF | HLA-A*23:01 | HLA-A*24:02 |  |  |
| YP_009724391.1 | ORF3a protein | 137 | 145 | NPLLYDANY | HLA-B*35:01 |  |  |  |
| YP_009724391.1 | ORF3a protein | 139 | 147 | LLYDANYFL | HLA-A*02:01 |  |  |  |
| YP_009724391.1 | ORF3a protein | 159 | 167 | PYNSVTSSI | HLA-A*24:02 |  |  |  |
| YP_009724391.1 | ORF3a protein | 180 | 193 | SEHDYQIGGYTEKW | HLA-B*44:02 | HLA-B*44:03 |  |  |
| YP_009724391.1 | ORF3a protein | 206 | 215 | YFTSDYYQLY | HLA-A*01:01 | HLA-A*01:01 | HLA-A*23:01 | HLA-A*24:02 |
| YP_009724391.1 | ORF3a protein | 211 | 219 | YYQLYSTQL | HLA-A*23:01 | HLA-A*24:02 |  |  |
| YP_009724391.1 | ORF3a protein | 220 | 233 | STDTGVEHVTFFIY | HLA-A*01:01 |  |  |  |
| YP_009724391.1 | ORF3a protein | 241 | 249 | EEHVQIHTI | HLA-B*44:02 | HLA-B*44:03 |  |  |
| YP_009724392.1 | envelope protein | 50 | 58 | SLVKPSFYV | HLA-A*02:01 |  |  |  |
| YP_009724392.1 | envelope protein | 57 | 65 | YVYSRVKNL | HLA-B*08:01 |  |  |  |
| YP_009724393.1 | membrane glycoprotein | 6 | 14 | GTITVEELK | HLA-A*11:01 |  |  |  |
| YP_009724393.1 | membrane glycoprotein | 11 | 20 | EELKKLLEQW | HLA-B*44:02 | HLA-B*44:03 |  |  |
| YP_009724393.1 | membrane glycoprotein | 15 | 23 | KLLEQWNLV | HLA-A*02:01 |  |  |  |
| YP_009724393.1 | membrane glycoprotein | 37 | 45 | FAYANRNRF | HLA-B*35:01 |  |  |  |
| YP_009724393.1 | membrane glycoprotein | 39 | 47 | YANRNRFLY | HLA-B*35:01 |  |  |  |
| YP_009724393.1 | membrane glycoprotein | 94 | 103 | SYFIASFRLF | HLA-A*23:01 | HLA-A*24:02 |  |  |
| YP_009724393.1 | membrane glycoprotein | 101 | 109 | RLFARTRSM | HLA-B*08:01 |  |  |  |
| YP_009724393.1 | membrane glycoprotein | 122 | 129 | VPLHGTIL | HLA-B*07:02 |  |  |  |
| YP_009724393.1 | membrane glycoprotein | 136 | 145 | SELVIGAVIL | HLA-B*40:01 |  |  |  |
| YP_009724393.1 | membrane glycoprotein | 148 | 156 | HLRIAGHHL | HLA-B*08:01 |  |  |  |
| YP_009724393.1 | membrane glycoprotein | 150 | 158 | RIAGHHLGR | HLA-A*03:01 |  |  |  |
| YP_009724393.1 | membrane glycoprotein | 166 | 176 | KEITVATSRTL | HLA-B*40:01 |  |  |  |
| YP_009724393.1 | membrane glycoprotein | 171 | 180 | ATSRTLSYYK | HLA-A*11:01 | HLA-A*01:01 | HLA-B*35:01 |  |
| YP_009724393.1 | membrane glycoprotein | 183 | 196 | ASQRVAGDSGFAAY | HLA-A*01:01 |  |  |  |
| YP_009724393.1 | membrane glycoprotein | 187 | 196 | VAGDSGFAAY | HLA-A*01:01 |  |  |  |
| YP_009724394.1 | ORF6 protein | 3 | 11 | HLVDFQVTI | HLA-A*02:01 |  |  |  |
| YP_009724394.1 | ORF6 protein | 36 | 44 | IIKNLSKSL | HLA-B*08:01 |  |  |  |
| YP_009724395.1 | ORF7a protein | 21 | 30 | QECVRGTTVL | HLA-B*40:01 |  |  |  |
| YP_009724395.1 | ORF7a protein | 40 | 49 | YEGNSPFHPL | HLA-B*40:01 |  |  |  |
| YP_009724395.1 | ORF7a protein | 44 | 56 | SPFHPLADNKFAL | HLA-B*07:02 |  |  |  |
| YP_009724395.1 | ORF7a protein | 63 | 75 | FAFACPDGVKHVY | HLA-B*35:01 |  |  |  |
| YP_009724395.1 | ORF7a protein | 69 | 77 | DGVKHVYQL | HLA-B*08:01 |  |  |  |
| YP_009724395.1 | ORF7a protein | 78 | 86 | RARSVSPKL | HLA-B*07:02 |  |  |  |
| YP_009724395.1 | ORF7a protein | 85 | 93 | KLFIRQEEV | HLA-A*02:01 |  |  |  |
| YP_009724395.1 | ORF7a protein | 94 | 102 | QELYSPIFL | HLA-B*40:01 |  |  |  |
| YP_009724396.1 | ORF8 protein | 32 | 42 | VVDDPCPIHFY | HLA-A*01:01 |  |  |  |
| YP_009724396.1 | ORF8 protein | 72 | 81 | QYIDIGNYTV | HLA-A*23:01 | HLA-A*02:01 |  |  |
| YP_009724396.1 | ORF8 protein | 105 | 118 | YEDFLEYHDVRVVL | HLA-B*40:01 | HLA-A*02:01 |  |  |
| YP_009724397.2 | nucleocapsid phosphoprotein | 45 | 53 | LPNNTASWF | HLA-B*35:01 |  |  |  |
| YP_009724397.2 | nucleocapsid phosphoprotein | 65 | 74 | KFPRGQGVPI | HLA-B*07:02 |  |  |  |
| YP_009724397.2 | nucleocapsid phosphoprotein | 75 | 87 | NTNSSPDDQIGYY | HLA-A*01:01 | HLA-B*35:01 |  |  |
| YP_009724397.2 | nucleocapsid phosphoprotein | 105 | 113 | SPRWYFYYL | HLA-B*07:02 |  |  |  |
| YP_009724397.2 | nucleocapsid phosphoprotein | 222 | 230 | LLLDRLNQL | HLA-A*02:01 | HLA-B*08:01 |  |  |
| YP_009724397.2 | nucleocapsid phosphoprotein | 266 | 274 | KAYNVTQAF | HLA-B*35:01 |  |  |  |
| YP_009724397.2 | nucleocapsid phosphoprotein | 289 | 301 | QELIRQGTDYKHW | HLA-B*44:02 |  |  |  |
| YP_009724397.2 | nucleocapsid phosphoprotein | 311 | 319 | ASAFFGMSR | HLA-A*11:01 |  |  |  |
| YP_009724397.2 | nucleocapsid phosphoprotein | 318 | 330 | SRIGMEVTPSGTW | HLA-B*44:02 |  |  |  |
| YP_009724397.2 | nucleocapsid phosphoprotein | 321 | 331 | GMEVTPSGTWL | HLA-B*44:02 | HLA-B*44:03 | HLA-B*40:01 |  |
| YP_009724397.2 | nucleocapsid phosphoprotein | 325 | 333 | TPSGTWLTY | HLA-B*35:01 |  |  |  |
| YP_009724397.2 | nucleocapsid phosphoprotein | 359 | 370 | AYKTFPPTEPK | HLA-A*11:01 | HLA-A*03:01 |  |  |
| YP_009724397.2 | nucleocapsid phosphoprotein | 395 | 403 | LPAADLDDF | HLA-B*35:01 |  |  |  |
| YP_009725255.1 | ORF10 protein | 2 | 11 | GYINVFAFPF | HLA-A*23:01 |  |  |  |
| YP_009725255.1 | ORF10 protein | 5 | 13 | NVFAFPFTI | HLA-A*02:01 |  |  |  |
| YP_009725255.1 | ORF10 protein | 8 | 16 | AFPFTIYSL | HLA-A*24:02 |  |  |  |

This 628-peptide array spans the USA-WA1/2020 strain of SARS-CoV-2 (Grifoni et al 2020, reference 9).
