# Supplementary material for: CHARM: COVID-19 Health Action Response for Marines–Association of antigen-specific interferon-gamma and IL2 responses with asymptomatic and symptomatic infections after a positive qPCR SARS-CoV-2 test
Source: PLoS One. 2022 Apr 7;17(4):e0266691. doi: 10.1371/journal.pone.0266691 (PMC8989306; doi:10.1371/journal.pone.0266691)
Supplement: S6 Table — CD4+ and CD8+ epitopes within each SARS-CoV-2 protein were predicted (reference 40), synthesized, and combined in the CD4+CD8 peptide pool. (DOCX) [file pone.0266691.s008.docx]

**Table S6: Distribution of predicted CD4+ and CD8+ epitopes within SARS-CoV-2 proteins**

|  | **Number of peptides predicted within protein** | | | | | | **Total** |
| --- | --- | --- | --- | --- | --- | --- | --- |
| **T cell type** | **S protein** | **N protein** | **M protein** | **E protein** | **NS protein** | **Other Proteins** |  |
| **Number CD4 Epitopes/protein** | 20 | 7 | 8 | 8 | 101 | 97 | 241 |
| **% CD4 Epitopes/Total** | 8.30 | 2.90 | 3.32 | 3.32 | 41.91 | 40.25 |  |
|  |  |  |  |  |  |  |  |
| **Number CD8 Epitopes/protein** | 86 | 13 | 15 | 2 | 227 | 285 | 628 |
| **% CD8 Epitopes/Total** | 13.69 | 2.07 | 2.39 | 0.32 | 36.15 | 45.38 |  |

CD4+ and CD8+ epitopes within each SARS-CoV-2 protein were predicted (52), synthesized, and combined in the CD4+CD8 peptide pool.
